# Defined Nylon Oligomers Enable Mechanistic Insight Into Enzymatic Polyamide Depolymerization

**DOI:** 10.1002/cssc.70693

**Published:** 2026-06-09

**Authors:** Sukmanita Dewi, Hendrik Puetz, Ulrich Schwaneberg, Laura Hartmann

**Affiliations:** ^1^ Institute of Macromolecular Chemistry, Albert‐Ludwigs‐University of Freiburg Freiburg Germany; ^2^ Institute of Biotechnology, RWTH Aachen University Aachen Germany; ^3^ Freiburg Materials Research Center, Albert‐Ludwigs‐University of Freiburg Freiburg Germany

**Keywords:** enzymatic polymer degradation, nylon oligomers, polyamide depolymerization, sequence‐defined oligomers, solid‐phase synthesis

## Abstract

The remarkable progress in enzymatic polyester depolymerization has demonstrated the potential of biocatalysis for sustainable polymer recycling. In contrast, enzymatic polyamide (PA) degradation remains poorly understood, largely due to the lack of defined and accessible model substrates. Here, we report a robust solid‐phase synthesis strategy for generating defined, monodisperse nylon‐6 and nylon‐6,6 oligomers with adjustable chain lengths. This approach enables the rapid and reproducible assembly of nylon oligomers that faithfully mimic the amide backbone of bulk polyamides while remaining soluble and analytically traceable. The resulting oligomer library provides a standardized set of substrates for screening and characterizing amidase activity, a class of enzymes whose substrate scope toward polyamides has not yet been systematically defined. As a proof of concept, we demonstrate the applicability of these model substrates using a representative amidase, revealing distinct chain length‐dependent conversion profiles that illustrate the potential of this approach for mechanistic investigations into enzyme–nylon interactions. Notably, the degradation patterns observed for the oligomers closely match those obtained for bulk nylon materials, underscoring their relevance as realistic surrogates for polymer depolymerization. Overall, our synthetic strategy thus bridges a critical gap between polymer chemistry and enzymatic screening, providing a foundation for the rational exploration of biocatalytic polyamide depolymerization.

## Graphical abstract

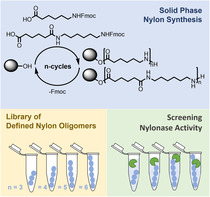

## 1 Introduction

The recent breakthroughs in enzymatic depolymerization of polyesters such as polyethylene terephthalate (PET) have demonstrated the power of biocatalysis for chemical recycling of synthetic polymers. Engineered cutinases and carboxylesterases have demonstrated highly efficient PET depolymerization under mild conditions, achieving high conversion levels under optimized reaction conditions [1, 2, 3]. More recently, similar biocatalytic strategies have been shown to enable efficient depolymerization of polycarbonates (PCs), with reported conversions exceeding 90% [4]. Inspired by these advances, attention has turned toward extending enzymatic depolymerization to polyamides (PAs), a major class of engineering polymers widely used in textiles, packaging, and automotive applications [5]. Among the various polyamides, nylon‐6 (PA6) and nylon‐6,6 (PA66) represent the most widely produced and industrially relevant types, together accounting for the majority of global PA demand [6, 7]. Despite the fact that proteins are themselves polyamides, degradation yields of nylons (PA6, PA66) have so far remained in the low single‐digit percentage range [8]. The translation of the above‐outlined concepts to polyamides has proven challenging primarily because extensive intermolecular hydrogen bonding between amide groups promotes dense chain packing and high crystallinity of nylon materials, which in turn severely limits enzymatic accessibility to amide bonds [9].

Despite these challenges, several microorganisms expressing amide‐hydrolyzing enzymes, such as amidases, proteases, and cutinases, have been reported to exhibit measurable polyamide (PA) biodegradation activity [7, 10, 11, 12], indicating that enzymatic nylon deconstruction may indeed be achievable. Building on this foundation, Bell and colleagues recently reported a landmark study screening over 40 candidate nylonases active on PA6 and quantifying eight soluble hydrolysis products by mass spectrometry, providing the most comprehensive assessment of nylonase activity to date, albeit with only low conversion levels observed [13]. Nevertheless, the number of enzymes that have been biochemically and structurally characterized in detail remains limited; only three hydrolases, NylA, NylB, and NylC from *Arthrobacter* sp., have been extensively studied as model systems for nylon degradation [7, 14, 15, 16, 17].

A major bottleneck in advancing this field is the lack of chemically defined, soluble model substrates that allow systematic evaluation of amidase activity. Most studies have relied on heterogeneous mixtures of nylon oligomers generated by partial hydrolysis or on insoluble nylon films and powders [13, 15, 18, 19, 20], which hinders quantitative kinetic analysis as well as meaningful comparison and engineering across enzyme systems. Alternative strategies rely on oligomers derived from controlled depolymerization of PA6 or PA66, followed by chromatographic separation [15, 21]. While chromatographic purification can provide access to individual oligomer species, these methods typically involve complex mixtures in the initial depolymerization step and require labor‐intensive isolation procedures that limit throughput and scalability for systematic enzymatic studies. The establishment of nylon oligomers with precise control over monomer composition, chain length, and end‐group structure—herein referred to as defined nylon oligomers—would therefore provide a foundation for reproducible enzymatic assays and accelerate the mechanistic understanding of polyamide‐active enzymes.

Solid‐phase synthesis (SPS) offers an attractive strategy for preparing such defined substrates. Since its introduction by Merrifield in 1963, SPS has become a cornerstone technique for the stepwise assembly of sequence‐defined, monodisperse macromolecules such as peptides, oligonucleotides, and oligosaccharides [22, 23, 24, 25, 26, 27]. By anchoring a growing chain to a functionalized resin, each coupling and deprotection step can be driven toward completion using excess reagents, while simple washing steps remove by‐products. This iterative approach provides precise control over monomer sequence and chain length, yielding monodisperse products without intermediate purification [24, 25, 26, 27].

While SPS is well established for biological polymers, its application to aliphatic polyamides has remained limited, likely because the hydrophobic nature and dense hydrogen‐bonding network of polyamide chains hinder resin swelling and reagent diffusion [9, 28]. In comparison, several solution‐phase strategies for monodisperse polyamides have been reported [29, 30, 31], yet these often face trade‐offs between chain length control, yield, and scalability. More recently, Franceschi et al. introduced a reversible adsorption solid support (RASS) method for the rapid synthesis of unnatural *γ*‐oligoamides (nylon‐4 type) using Fmoc chemistry on a soluble support [32]. In parallel, Tharayil et al*.* developed a scalable, solution‐phase protocol for monodisperse nylon‐4, nylon‐6, and hybrid nylon‐4/6 oligomers using a group‐assisted purification (GAP) strategy employing phosphonate‐functional soluble supports [33]. Despite these advances, the synthesis of defined aliphatic polyamides that faithfully reproduce the *ε*‐ and *α*,*ω*‐linked backbones of nylon‐6 (PA6) and nylon‐6,6 (PA66) remains challenging. Soluble‐support approaches require specialized monomers and elevated temperatures, while conventional solution‐phase coupling often lacks the reproducibility and throughput necessary for systematic preparation of multiple chain lengths.

To overcome these limitations, we developed a robust SPS protocol that enables rapid and reproducible assembly of monodisperse PA6 and PA66 oligomers under mild, room‐temperature conditions. In contrast to previously reported RASS and GAP strategies [32, 33], our approach employs commercially available or readily synthesized Fmoc‐protected *ω*‐amino acid monomers, standard peptide‐coupling reagents, and conventional solid supports. This provides a universal, accessible, and reproducible route to true nylon‐type oligomers that preserve the structural fidelity of industrial polyamides while remaining soluble and analytically traceable.

Investigating both PA6‐ and PA66‐type oligomers further enables systematic evaluation of how variations in polyamide structure influence enzymatic recognition and hydrolysis. Finally, we demonstrate that the reactivity trends observed with the synthetic oligomers mirror those obtained for bulk nylon materials by applying our system to the engineered NylC variant, NylC‐HP [17], underscoring the relevance of these oligomers as realistic model substrates. This work thus establishes a practical and versatile route to nylon model compounds and provides the foundation for systematic screening and mechanistic studies of enzymatic polyamide depolymerization.

## 2 Results and Discussion

### 2.1 Synthesis of Defined Nylon Oligomers

For the synthesis of nylon‐type oligomers, Wang resin was selected as the solid support owing to its acid‐labile linker [34], which allows cleavage under mild acidic conditions to yield C‐terminal carboxylic acids structurally analogous to the chain ends of bulk polyamides. The resin also exhibited favorable swelling in polar aprotic solvents such as DMF, thereby facilitating reagent diffusion even for hydrophobic aliphatic monomers [35]. This choice ensured efficient coupling throughout chain elongation and produced oligomers with terminal functionalities closely resembling those of conventional nylons.

**SCHEME 1:**
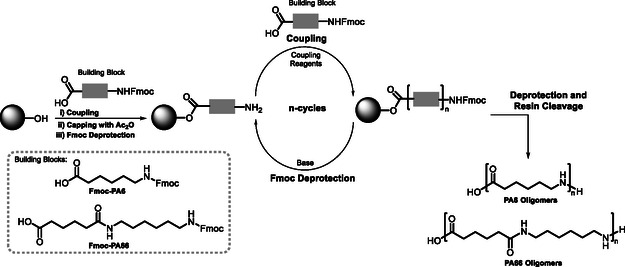
General overview of SPS of defined nylon oligomers. SPS = Solid‐phase synthesis.

The synthetic workflow is shown in Scheme 1. Fmoc‐protected *ω*‐amino acid derivatives were employed as activated building blocks: commercially available Fmoc‐6‐aminohexanoic acid (Fmoc‐PA6) and a synthetic Fmoc‐protected PA66 (Fmoc‐PA66) monomer (synthetic details in the Supporting Information (SI)). Oligomer growth proceeded through iterative cycles of amide coupling and Fmoc deprotection to regenerate the terminal amine. To prevent side reactions and ensure monodispersity, unreacted hydroxyl groups on the resin were capped after the first coupling step using acetic anhydride, effectively blocking residual reactive sites and preventing uncontrolled chain initiation or branching during subsequent cycles [36]. The iterative coupling−deprotection sequence was repeated until the desired degree of polymerization (*n* = 2−7) was achieved, and the defined oligomers were then cleaved from the resin under acidic conditions to afford the corresponding homopolyamide PA6_*n*_ and alternating copolyamide PA66_*n*_ oligomers.

To identify optimal conditions for aliphatic amide formation, a series of coupling reagents and bases were evaluated in the synthesis of a PA6 dimer (Table 1). Reactions were conducted at room temperature in DMF, and isolated crude yields and product purities were determined by gravimetry and reverse‐phase HPLC (RP‐HPLC) analysis, respectively. Because crude mass recoveries occasionally exceeded 100%, a common occurrence in SPS due to water uptake and salt association typical of hygroscopic amide oligomers, purity‐corrected yields (Table 1) are therefore reported to more accurately reflect synthetic efficiency.

**TABLE 1:** Evaluation of coupling reagents for the SPS of PA6 dimer (*n* = 2).

Coupling solvent	Coupling reagent	Base	Crude mass recovery^a^	Relative purity^b^	Corrected yield^c^
DMF	5 eq DIC^d^	0.1 eq DMAP^e^	122%	6%	7%
DMF	5 eq DIC/5 eq HOBt^f^	0.1 eq DMAP	52%	98%	51%
DMF	5 eq PyBOP^g^	10 eq DIPEA^h^	108%	92%	99%
DMF	3.3 eq PyBOP/1.7 eq HOBt	10 eq DIPEA	51%	84%	43%
DMF	5 eq MSNT^i^	7.5 eq MeIm^j^	105%	95%	100%
0.3 M LiCl in DMF	5 eq MSNT	7.5 eq MeIm	103%	98%	100%

a Isolated crude product obtained after resin cleavage and freeze‐drying; values exceeding 100% are attributed to residual solvent or salt content.

b Relative purity determined by integration of the RP‐HPLC/UV signal at 205 nm.

c Purity‐corrected yield calculated as (crude mass recovery × relative purity)/100.

d DIC = *N*,*N′*‐diisopropylcarbodiimide.

e DMAP = 4‐(*N*,*N*‐dimethylamino)pyridine.

f HOBt = 1‐hydroxybenzotriazole.

g PyBOP = benzotriazol‐1‐yl‐oxytripyrrolidinophosphonium hexafluorophosphate.

h DIPEA = *N*,*N′*‐diisopropylethylamine.

i MSNT = 2,4,6‐mesitylenesulfonyl‐3‐nitro‐1,2,4‐triazole.

j MeIm = 1‐methylimidazole.

**FIGURE 1:**
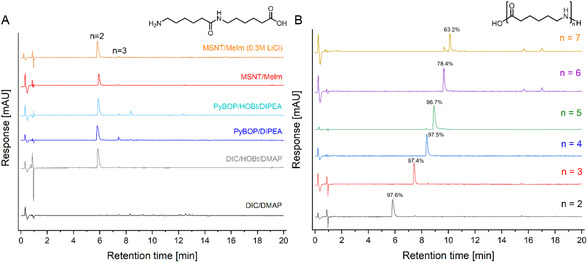
Evaluation of coupling conditions during the SPS of PA6 dimer on Wang resin (A) and synthesis of defined PA6 oligomers (*n* = 2−7) under optimized conditions (MSNT/MeIm in 0.3 M LiCl/DMF) without further purification (B). RP‐HPLC chromatograms were recorded using a linear gradient from 1 to 50% acetonitrile over 15 min with UV detection at 205 nm. Relative purities were determined by peak integration. SPS = Solid‐phase synthesis.

The carbodiimide‐based system [36] *N*,*N′*‐diisopropylcarbodiimide (DIC) and 4‐(*N*,*N*‐dimethylamino)pyridine (DMAP) produced extensive side reactions, including the formation and rearrangement of *O*‐acylisourea intermediates to *N*‐acylureas, [37] resulting in a very low product purity (Figure 1A). Such side reactions are commonly observed in carbodiimide‐mediated coupling of hydrophobic aliphatic substrates, where limited resin swelling and prolonged contact with reactive intermediates promote undesired acyl transfer pathways [38]. Addition of 1‐hydroxybenzotriazole (HOBt) [25, 38] effectively suppressed these side reactions and improved purity to 98%, although conversion remained incomplete. In contrast, phosphonium‐based activation [26, 27] using benzotriazol‐1‐yl‐oxytripyrrolidinophosphonium hexafluorophosphate (PyBOP) and thioimidate‐based activation [39, 40] with 2,4,6‐mesitylenesulfonyl‐3‐nitro‐1,2,4‐triazole (MSNT) yielded substantially cleaner chromatograms dominated by the target product peak (Figure 1A). The PyBOP/DIPEA system afforded excellent yield (99%) and good purity (92%), whereas inclusion of HOBt reduced conversion, likely due to competing side reactions with the activated intermediate. Among all systems tested, the MSNT/MeIm provided the highest yield and purity in DMF. Remarkably, addition of 0.3 M Lithium chloride (LiCl) to the solvent further improved both resin swelling and solubilization of the activated intermediates, yielding near‐quantitative conversion (>99%) and excellent purity (98%). The beneficial effect of LiCl is attributed to its ability to disrupt intra‐ and intermolecular hydrogen bonding between growing polyamide chains, thereby reducing adhesion of oligomers and reactive intermediates to the resin surface and facilitating efficient removal of residual side products [27, 28, 41].

The impact of LiCl is further illustrated in Figure S11, which compares syntheses performed with and without LiCl during coupling of PA6 trimers to hexamers. In the absence of LiCl, reactive mesitylenesulfonyl‐activated intermediates (Ahx‐OMs) formed from excess MSNT and monomer residues tend to adhere strongly to the resin because of their limited solubility and high polarity. Representative structures of such reactive intermediates are shown in Figure S12. These species can persist through subsequent coupling cycles, leading to partial over‐ or underacylation and reduced product homogeneity. Furthermore, starting from the pentamer, Fmoc carryover was also detected as Fmoc‐PA6_5_ impurities, likely arising from incomplete deprotection at sterically hindered or poorly swollen resin sites during later cycles. Addition of 0.3 M LiCl significantly improved resin swelling and the solubility of both Ahx‐OMs intermediates and hydrophobic oligomeric impurities, producing cleaner reaction profiles across all chain lengths.

Thus, MSNT/MeIm in 0.3 M LiCl/DMF was chosen as the standard coupling system for subsequent syntheses of PA6 trimers up to heptamers. Under these optimized conditions, PA6 oligomers up to the pentamer were obtained in 99% overall yield and >95% purity without further purification (Figure 1B). Minor signals corresponding to n – 1 and *n* + 1 oligomer species were occasionally detected and are attributed to trace dimer impurities present in the commercial Fmoc‐PA6 monomer (see SI, Figure S9). For longer sequences, particularly the hexamer and heptamer, small amounts of insoluble oligoamide side products remained attached to the resin even after LiCl washing, although their abundance was markedly lower than in reactions conducted without LiCl. The coupling to form the heptamer represented the practical upper limit of the current protocol, as a residual hexamer peak (1:5 ratio) persisted in the final product mixture. This limitation likely reflects the combined steric and solvation constraints inherent to longer aliphatic chains rather than intrinsic coupling failure. Hexamer and heptamer products were further purified by preparative C18 reversed‐phase chromatography to remove residual side products and obtain analytically pure materials (Figure S21‐S25).

**FIGURE 2:**
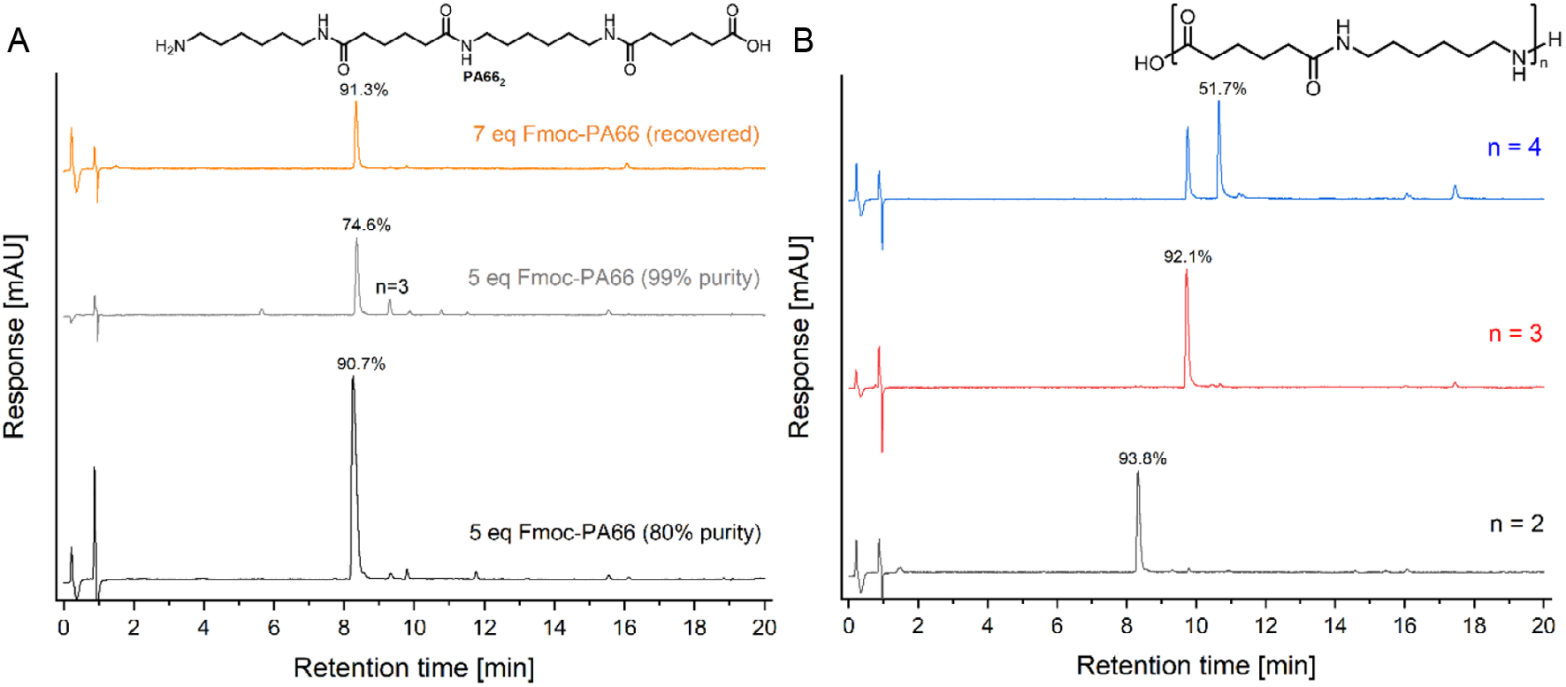
Effect of building‐block purity on product quality during the SPS of PA66 dimer using MSNT/MeIm standard conditions (A) and synthesis of defined PA66 oligomers (*n* = 2−4) under optimized conditions (MSNT/MeIm in 0.3 M LiCl/DMF) without further purification (B). RP‐HPLC chromatograms were recorded using a linear gradient from 1 to 50% acetonitrile over 15 min with UV detection at 205 nm. Relative purities were determined by peak integration. SPS = Solid‐phase synthesis.

The optimized coupling system was subsequently applied to the synthesis of PA66 oligomers, using the Fmoc‐protected PA66 building block derived from adipic acid and hexamethylenediamine (synthetic details in SI). Fmoc‐PA66 was obtained in moderate yield (58%) and ≈80% purity. RP‐HPLC analysis revealed a significant secondary peak at t_R_ ≈ 15.2 min (Figure S8), consistent with Fmoc‐adipate‐type side products formed during activation or coupling steps of monomer preparation. To evaluate whether further purification was required, the unrefined monomer mixture was directly employed in SPS of the PA66 dimer under standard MSNT/MeIm conditions. Remarkably, the desired product was obtained in >90% purity, albeit with a low overall yield (Table 2, entry 1; Figure 2A). In contrast, when the purified monomer was used under identical conditions, a larger number of side products were observed (entry 2; Figure 2A), indicating that the impurities present in the unrefined monomer did not interfere with coupling and may even exert a moderating effect that enhances selectivity. This counterintuitive trend can be explained by subtle differences in reaction microenvironment. The Fmoc‐adipate‐type by‐products likely act as inert, weakly reactive cocomponents that provide slight steric and solvation buffering, thereby moderating local monomer concentration and suppressing unwanted side reactions. Upon their removal, the purified monomer becomes more reactive and locally concentrated, favoring self‐condensation or acyl‐exchange pathways that diminish overall selectivity. Consequently, the “cleaner” monomer paradoxically yielded a more complex product profile and lower overall yield.

**TABLE 2:** Evaluation of coupling conditions for the SPS of PA66 dimer (*n* = 2).

Entry	Building block		Coupling reagent	Coupling time	Product	
	Equiv	Purity^a^			Relative purity^b^	Corrected yield^c^
1	5 eq	80%	5 eq MSNT/7.5 eq MeIm	3 h	91%	32%
2	5 eq	99%	5 eq MSNT/7.5 eq MeIm	3 h	75%	17%
3	7 eq	80%	6 eq MSNT/8 eq MeIm	3 h	94%	69%
4	8 eq	80%	7 eq MSNT/10 eq MeIm	3 h	94%	96%
5	7 eq	80%	6 eq MSNT/8 eq MeIm	2 × 3 h	94%	100%
6	7 eq	recover	6 eq MSNT/8 eq MeIm	3 h	91%	100%

a Purity determined by integration of the RP‐HPLC/UV signal using a 5–95% acetonitrile gradient at 214 nm.

b Relative purity determined by integration of the RP‐HPLC/UV signal using a 1–50% acetonitrile gradient at 205 nm.

c Purity‐corrected yield calculated as (crude product recovery × relative purity)/100.

As shown in Table 2, increasing the number of equivalents of unrefined monomer markedly improved the yield, with 7–8 equivalents affording near‐quantitative conversion while maintaining high purity. Performing double coupling cycles gave only marginal improvement. Moreover, the Fmoc‐PA66 building block could be regenerated efficiently by acid precipitation with 2.5 M HCl (Figure S8), and reuse of the recovered monomer afforded a high‐purity PA66 dimer comparable to that from fresh material (entry 6; Figure 2A). This demonstrates both the recyclability of the Fmoc‐PA66 building block and the broad applicability of the optimized MSNT/MeIm system to more rigid *α*,*ω*‐linked nylon backbones. The optimal conditions for PA66 oligomer synthesis were therefore established as 8 equivalents of building block, 7 equivalents of MSNT, and 10 equivalents of MeIm (Table 2, entry 4) and subsequently applied to the synthesis of PA66 trimer and tetramer (Figure 2B).

The PA66 trimer was obtained in >90% purity, confirming efficient coupling under these conditions, although small signals corresponding to mesitylenesulfonyl‐activated side products (PA66_*x*_‐OMs) and minor Fmoc‐carryover species were detectable. Upon extending the synthesis to the tetramer, the chromatogram displayed two principal peaks in an approximate 1:1.5 ratio, with the secondary peak corresponding to residual PA66_3_ from incomplete chain elongation, while later‐eluting side products became substantially more pronounced. The accumulation of these species correlates with increasing chain length and reflects reduced resin swelling and slower reagent diffusion in longer, increasingly hydrophobic polyamide sequences. Consequently, the PA66 tetramer represents the practical upper limit of efficient chain elongation under the present solid‐phase conditions. The tetramer product was further purified by preparative C18 reversed‐phase chromatography to afford analytically pure material (Figure S31‐S32).

Taken together, these results demonstrate that steric congestion, incomplete chain extension, and residual activated intermediates are the key factors currently limiting further growth in solid‐phase nylon synthesis. Nevertheless, the developed protocol enables the rapid and reproducible preparation of monodisperse PA6 and PA66 oligomers in high purity, without additional purification, up to chain lengths of five and three amide units, respectively. Both oligomer series retained full sequence fidelity and showed no evidence of cyclic by‐products, underscoring the reliability of solid‐phase assembly even for highly hydrogen‐bonded polyamide backbones. The resulting oligomers are structurally defined, soluble, and analytically tractable, making them ideal model substrates for enzymatic hydrolysis and mechanistic investigations of polyamide‐degrading enzymes.

### 2.2 Enzymatic Hydrolysis of Nylon

#### 2.2.1 Hydrolysis of Defined Oligomers

With access to a library of chemically defined PA6 and PA66 oligomers, we next investigated their suitability as model substrates for enzymatic hydrolysis. Their monodisperse nature and known chain lengths provide a unique opportunity to systematically evaluate how backbone composition and degree of polymerization influence enzymatic activity. Previous studies on nylon degradation have largely relied on ill‐defined oligomer mixtures or bulk polymer films, which obscure mechanistic interpretation and complicate quantitative analysis [13, 19]. In contrast, the defined oligomers obtained from SPS enable precise monitoring of individual cleavage events, facilitating direct comparison of enzyme selectivity and turnover across different substrate architectures.

**FIGURE 3:**
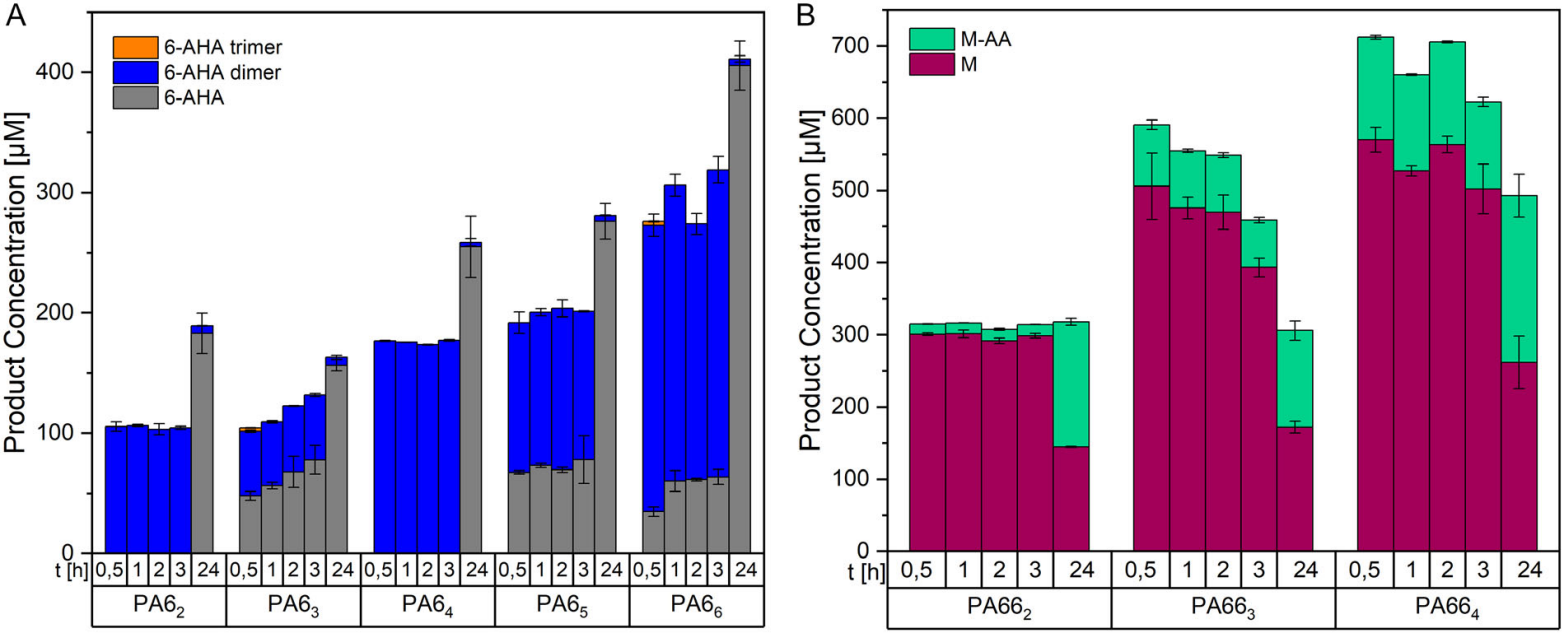
Enzymatic hydrolysis of defined nylon oligomers by NylC‐HP. Time‐resolved product formation from (A) PA6_*n*_ (*n* = 2−6) and (B) PA66_*n*_ (*n* = 2−4) substrates. Reactions contained 1 µM enzyme and 100 µM substrate, incubated at 70°C for 24 hr. All reactions were performed in triplicate (*n* = 3); error bars represent the standard deviation of replicate measurements.

The primary objective of this study was to establish and validate defined nylon oligomers as mechanistic substrates rather than to perform a broad enzyme screen, hence we employed a single representative amidase, the engineered variant NylC‐HP (NylC_p2_‐TS^F134W/D304M/R330A^) [17]. NylC‐type enzymes are among the best‐studied nylonases and exhibit robust activity toward both PA6 and PA66 fragments [17, 18]. They are known to cleave internal amide bonds in aliphatic polyamides, particularly within PA6 oligomers [15], making NylC‐HP a suitable benchmark catalyst for probing chain length‐dependent reactivity. Using purified NylC‐HP (see SI for expression and purification), we conducted hydrolysis reactions with PA6_*n*_ and PA66_*n*_ oligomers of defined chain lengths (*n* = 2–7 and *n* = 2–4, respectively). Reactions were performed in 100 mM potassium phosphate buffer (pH 7.5, 150 mM NaCl), containing 1 μM enzyme and 100 µM substrate. Reaction progress was monitored by RP‐HPLC and confirmed by electrospray ionization mass spectrometry (ESI‐MS), allowing quantification of cleavage products and identification of hydrolysis patterns over 24 h. Control incubations without enzyme showed no detectable substrate conversion (see SI Figure S39 for exemplary spectra). This experimental setup directly links molecular structure to enzymatic response, providing mechanistic insight into amidase specificity and substrate recognition (Figure 3).

The nylon‐6 dimer (PA6_2_) remained intact during the initial incubation phase and only slowly converted to 6‐aminohexanoic acid (6‐AHA) over 24 h, indicating that NylC‐HP has limited activity toward the shortest amide oligomers. In contrast, PA6_3_ was completely hydrolyzed within 1 h, yielding both dimer and monomer products. The tetramer (PA6_4_) displayed a distinct cleavage pattern: two dimer fragments as the predominant products, suggesting preferential hydrolysis at the central amide bond. These dimers persisted throughout the assay, implying slower turnover beyond the initial scission. The pentamer (PA6_5_) was rapidly fragmented into dimer and trimer intermediates, which subsequently degraded into monomeric and dimeric species. The hexamer (PA6_6_) exhibited immediate release of 6‐AHA, consistent with a primary cut near the chain center yielding two trimers. Collectively, these results indicate that NylC‐HP is catalytically competent toward nylon oligomers once a minimal chain length is reached, and the enzymatic activity or stability is not the primary limiting factor under the conditions tested [42]. Instead, the efficiency of depolymerization appears to be governed by substrate accessibility and productive binding of the enzyme to amide bonds, highlighting the need for improved or engineered enzymes capable of overcoming these structural constraints characteristic of polyamide substrates.

Interestingly, PA6_6_ showed only partial solubility in aqueous buffer, forming dispersions that likely restricted complete enzymatic access. This solubility threshold is consistent with the expected increase in crystallinity and hydrogen bonding density at higher chain lengths [43]. Short oligomers of this length are already sufficiently long to establish intermolecular hydrogen‐bond networks analogous to those in bulk nylon, resulting in markedly reduced solubility even at elevated temperature [44]. The transition from soluble, monodisperse oligomers to semicrystalline, polymer‐like material thus occurs between PA6_6_ and PA6_7_, marking the upper boundary of the soluble substrate range (see SI, Figure S40). Although this limits quantitative kinetic analysis, inclusion of these longer oligomers remains valuable for approximating enzyme activity near the soluble–insoluble boundary. The corresponding assay with PA6_7_ is provided in the SI (Figure S41) and displays consistent fragmentation patterns.

In contrast, NylC‐HP activity toward PA66 oligomers followed a different trend. Upon incubation, all PA66 substrates were rapidly cleaved to yield the monomeric unit hexamethylenediamine–adipic acid (M) as the dominant soluble product, accompanied by smaller quantities of the diacid‐terminated fragment adipic acid–hexamethylenediamine–adipic acid (M–AA) (Figure 3B). The monomer accumulated quickly during the initial phase, consistent with direct hydrolysis of internal amide bonds to release M. The minor formation of M–AA reflects asymmetric cleavage events. Over extended incubation (24 h), the concentration of M decreased while M–AA continued to increase, suggesting that M undergoes further hydrolysis into smaller species. According to established degradation pathways for PA66, this process ultimately yields adipic acid (AA) and hexamethylenediamine (HMDA) [17, 19, 21, 45], although these final products were not directly detected under the applied analytical conditions. In contrast, M–AA remains largely resistant to enzymatic attack due to steric and electrostatic hindrance from its terminal carboxylate groups.

Starting from the PA66 trimer (PA66_3_), the substrate was not fully soluble under assay conditions, forming dispersions that did not clear upon gentle heating or equilibration. This limited solubility reflects the higher hydrogen‐bond density and nascent crystallinity of PA66 oligomers, which cross the oligomer‐to‐polymer solubility threshold earlier than the corresponding PA6 series [43]. Consequently, PA66_3_ and PA66_4_ behave as heterogeneous substrates in the enzymatic assays, and the observed product distributions must be interpreted as surface‐limited hydrolysis events rather than homogeneous kinetic transformations.

Enzyme‐scaling experiments with the PA66_4_ confirmed that both monomer formation and its subsequent consumption are directly proportional to enzyme concentration (Figure S42). At higher NylC‐HP loadings, substrate turnover was rapid, with near‐complete monomer formation and depletion within shorter time frames. At lower enzyme concentrations, however, the monomer accumulated to a higher apparent steady‐state level, reflecting slower secondary degradation. Meanwhile, the M–AA fragment accumulated progressively in all cases, indicating its kinetic stability as a dead‐end product of PA66 hydrolysis. Although inhibition was not assessed systematically, no apparent substrate or product inhibition was observed from the time‐course profiles under the applied conditions and product concentrations reached in this study. Thus, the observed product distributions likely reflect intrinsic cleavage preferences and differential turnover of terminal fragments rather than inhibition effects.

Taken together, these results confirm that NylC‐HP catalyzes rapid, endo‐type initial cleavage of both PA6 and PA66 oligomers, followed by slower exo‐type hydrolysis of terminal fragments. The preferential accumulation of M–AA demonstrates that terminal diacid moieties substantially reduce enzymatic accessibility or susceptibility to hydrolysis, leading to incomplete degradation. The solubility thresholds observed for PA6_6_ and PA66_3_ mark the onset of polymer‐like behavior, bridging soluble‐oligomer kinetics and bulk polymer degradation.

#### 2.2.2 Hydrolysis of Bulk Polyamide Substrates

To evaluate whether the observed chain length trends extend to bulk polyamide materials, we next examined the hydrolysis of PA6 film (thickness 0.2 mm; Goodfellow GmbH, Hamburg, Germany) and PA66 granules (diameter 3 mm; Goodfellow GmbH, Hamburg, Germany) under the same buffer and temperature conditions employed for the oligomer assays. Reactions were conducted using 1 μM NylC‐HP and 2 mg PA6 film (0.67 wt% substrate, 0.5 mM enzyme g^−1^ PA6) and 3.2 µM NylC‐HP and 11 mg PA66 granules (2.16 wt% substrate, 0.3 mM enzyme g^−1^ PA66), respectively. These enzyme loadings were intentionally selected to exceed previously reported values by Bell et al*.* in order to assess whether increased enzyme concentration could enhance product release [13]. Accordingly, the goal of these experiments was not to achieve high conversion but to determine whether the reaction pathways and product profiles observed for the PA6 and PA66 oligomers persisted when NylC‐HP acted on semicrystalline polymer surfaces.

**FIGURE 4:**
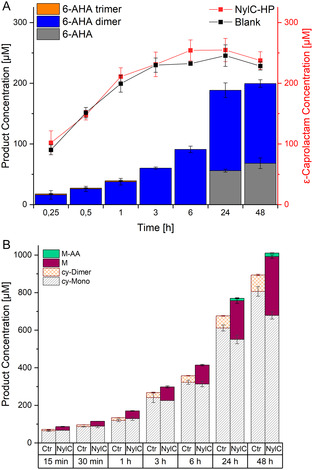
Enzymatic hydrolysis of bulk polyamide materials by NylC‐HP. (A) Product distribution from nylon‐6 film incubated with 1 µM enzyme. (B) Product distribution from nylon‐6,6 granules incubated with 3.2 µM enzyme. Reactions were performed in 100 mM potassium phosphate buffer (pH 7.5, 150 mM NaCl) at 70°C for 48 hr. Ctr (blank): reactions without enzyme, NylC: reactions containing enzyme. All assays were carried out in triplicate (*n* = 3); error bars represent the standard deviation of replicate measurements.

For PA6 film, *ε*‐caprolactam was detected at comparable levels in both enzyme‐containing and blank samples throughout the time course (see SI Figure S43 for exemplary spectra), confirming that NylC‐HP does not catalyze lactam ring opening under these neutral conditions as previously observed and that the lactam signal originates from preexisting low‐molecular‐weight species in the material rather than from enzymatic or spontaneous hydrolysis of the polymer backbone [18, 46]. This observation aligns with the fact that polyamide manufacturing typically results in the entrainment of residual monomeric starting materials and cyclic oligomers, which can leach out as water permeates the polymer matrix [46, 47]. In the presence of NylC‐HP, only trace amounts of linear 6‐AHA species were released, with the dimer as the predominant soluble product and small quantities of monomer appearing after prolonged incubation (Figure 4A). The total concentration of 6‐AHA equivalents remained around 200 µM, in line with the observations of Bell et al*.*, who reported similarly low release levels (<300 µM) over 10 days across a range of reaction conditions [13]. Consistent with these observations, increasing enzyme loading did not result in a substantial increase in overall depolymerization, indicating that hydrolysis of semicrystalline polyamides is primarily limited by substrate accessibility rather than enzyme concentration.

In the case of PA66 granules, blank reactions contained both cyclic monomer and cyclic dimer species (Figure 4B and Figure S44), reflecting the presence of extractable low‐molecular‐weight species analogous to those observed for PA6. Upon incubation with NylC‐HP, the enzyme very slowly hydrolyzed the cyclic monomer but readily opened the cyclic dimer to yield the linear monomeric unit M (hexamethylenediamine–adipic acid). After extended incubation (≥24 h), minor amounts of the diacid‐terminated fragment M–AA were also observed. This selectivity for cyclic dimer over cyclic monomer can be rationalized by conformational accessibility. The larger macrocycle exposes less intramolecularly hydrogen‐bonded amide bonds that can more easily enter the NylC‐HP active site, whereas the smaller cyclic monomer is more conformationally constrained and less reactive. The product distribution therefore mirrors the mechanistic behavior observed for the linear PA66 oligomers, in which NylC‐HP preferentially performs endo‐type cleavage on segments above a minimal length threshold.

Across both bulk substrates, the total amount of soluble monomeric products released after 48 h corresponded to only minimal conversion of total amide bonds, amounting to less than 1% for both PA6 and PA66 (≈0.62% and 0.38%, respectively), consistent with strongly surface‐limited hydrolysis of semicrystalline polyamides. Similar limitations have also been reported for the other polymer systems such as PET, where efficient enzymatic depolymerization typically requires pretreatment (e.g., thermal or mechanical amorphization) to increase substrate accessibility [48, 49, 50]. These findings highlight that effective enzymatic degradation of bulk nylon materials will likewise depend on appropriate pretreatment strategies. Despite these low overall yields, the product profiles match those observed for the corresponding PA6 and PA66 oligomers, demonstrating that NylC‐HP follows the same mechanistic pathway when acting on polymer surfaces as when acting on monodisperse oligomers in solution.

## 3 Conclusion

In this work, we established a robust solid‐phase synthetic route for the preparation of defined nylon‐6 and nylon‐6,6 oligomers with precise chain lengths and high purity. These monodisperse, chemically uniform substrates bridge the gap between small‐molecule model compounds and bulk polymers, providing an unprecedented level of structural definition for mechanistic studies of enzymatic nylon hydrolysis.

Using the engineered amidase NylC‐HP (NylC_p2_‐TS^F134W/D304M/R330A^), we demonstrated that both PA6 and PA66 oligomers undergo rapid, endo‐type cleavage of internal amide bonds, followed by slower exo‐type hydrolysis of terminal fragments. The enzyme displayed a clear chain length threshold for efficient turnover, with oligomers of three or more amide units being rapidly processed, while shorter or diacid‐terminated substrates remained largely resistant. Comparison across the PA6 and PA66 series revealed that backbone composition and end‐group chemistry strongly influence both solubility and enzymatic accessibility, ultimately shaping the observed product distributions. The onset of reduced solubility at PA6_6_ and PA66_3_ further marks the transition from homogeneous solution‐phase hydrolysis to surface‐limited catalysis characteristic of semicrystalline polymers.

Extending the assays to bulk PA6 films and PA66 granules showed that, despite very low overall conversion, the resulting product profiles closely mirrored those obtained with soluble oligomers. This consistency confirms that the mechanistic trends identified at the oligomer level remain relevant at polymer surfaces and underscores the value of defined nylon oligomers as model substrates for dissecting structure–reactivity relationships in polyamide degradation.

Notably, other canonical nylon hydrolases, such as NylA and NylB, remain similarly limited in terms of overall degradation efficiency, highlighting a broader need for new or improved nylonases. In this context, the defined oligomer platform presented here provides a versatile basis for mechanistic studies, enzyme benchmarking, and the discovery of polyamide‐hydrolyzing enzymes with enhanced depolymerization performance. By enabling systematic analysis of chain length‐dependent reactivity, backbone effects, and end‐group influences, this platform establishes a molecular framework for the rational design of improved polyamide hydrolases and supports ongoing efforts toward effective biocatalytic depolymerization and recycling of synthetic polyamides.

## 4 Experimental Section

### 4.1 Solid‐Phase Synthesis of Nylon Oligomers

**Resin selection**. Polystyrene PHB−Wang resin was used as the solid support for all solid‐phase syntheses. All procedures were performed on a 0.2 mmol scale unless otherwise noted, with washing steps conducted using 6 mL of the indicated solvent. Prior to coupling, the resin was swollen in dichloromethane (DCM) in a 10 mL polypropylene syringe reactor equipped with a polyethylene frit (Multisyntech GmbH, Witten, Germany). The resin was shaken twice for 30 min to allow sufficient swelling, then washed thoroughly with *N*,*N*‐dimethylformamide (DMF) (10x) to remove residual contaminants and ensure complete solvent exchange.

**Coupling and Fmoc‐deprotection.** For each coupling step, the activated building block solution was prepared as follows. Fmoc‐6‐aminohexanoic acid (Fmoc‐PA6; 5 equiv) or Fmoc‐PA66 (8 equiv) was dissolved in 5 mL DMF containing 0.3 M LiCl. Coupling reagents 1‐(mesitylsulfonyl)‐3‐nitro‐1,2,4‐triazole (MSNT; 5 equiv for PA6, 7 equiv for PA66) and 1‐methylimidazole (MeIm; 7.5 equiv for PA6, 10 equiv for PA66) were added, and the mixture was flushed with nitrogen, sealed, and shaken for 5 min to ensure complete dissolution and preactivation. The coupling solution was then transferred to the preswelled resin, and the coupling reaction was performed with continuous shaking for 3 h at room temperature.

For the first coupling cycle only, a second coupling step was performed under identical conditions, followed by overnight shaking to ensure complete reaction with surface hydroxyl groups. After this initial attachment step, the resin was washed five times with DMF and five times with DCM, and any remaining hydroxyl groups were capped with a 1:1 mixture of acetic anhydride and pyridine (2 × 1 h). This double‐coupling and capping procedure was applied only during the initial attachment step; all subsequent cycles involved only a single coupling step followed by Fmoc deprotection. After capping, the resin was washed 10 times with DCM and 10 times with DMF.

Fmoc deprotection was carried out by treating the resin with 25% piperidine in DMF containing 0.3 M LiCl for 20 min, followed by a second 10 min treatment. The resin was then washed thoroughly with DMF (15x). The coupling‐deprotection cycle was repeated iteratively until the desired chain length (*n* = 2−7) was achieved.

**Recovery of excess building blocks.** Excess building block solution was recovered after each coupling step. Following coupling, the remaining reaction mixture was poured directly into a 2.5 M hydrochloric acid (HCl) solution, which immediately produced a white precipitate while the coupling reagents remained soluble in the aqueous phase. The suspension was allowed to stand until precipitation was complete, after which the solid was collected by filtration and dried under vacuum to afford the Fmoc‐protected building blocks (Fmoc‐PA66 and Fmoc‐PA6) with purities of 85% and 96%, respectively (see Figures S8 and S10).

**Cleavage and purification.** The resin was first conditioned by washing five times with DCM. Cleavage from the solid support was then performed by treating the resin with 5 mL of an acidic mixture of trifluoroacetic acid (TFA), triisopropylsilane (TIPS; carbocation scavenger), and DCM (95:2.5:2.5, v/v/v) for 1 h at room temperature. To maximize yield and ensure complete detachment from the resin, the cleavage step was repeated once. The combined filtrates were concentrated under a nitrogen stream until all volatile components had evaporated. The crude product was dissolved in Milli‐Q water and lyophilized to afford the isolated oligomers. PA6 dimers and trimers were obtained as yellow oils, whereas longer PA6 oligomers and all PA66 oligomers were isolated as white powders. For higher oligomers (PA6 hexamer and heptamer, and PA66 tetramer), further purification was performed by preparative C18 reverse‐phase chromatography to obtain analytically pure materials (Figures S21–S24 and S29).

### 4.2 Enzymatic Hydrolysis Assay

**Nylon oligomer assays.** Enzymatic hydrolysis was carried out using purified NylC‐HP. Defined PA6_*n*_ and PA66_*n*_ oligomers (*n* = 2–7 and *n* = 2–4, respectively) were incubated at a final concentration of 100 µM in 100 mM potassium phosphate buffer (pH 7.5) containing 150 mM NaCl. Reactions were initiated by the addition of 1 µM enzyme and incubated at 70°C in a Thermomixer C (Eppendorf SE, Hamburg, Germany) with shaking at 400 rpm. Aliquots were collected at defined time points over 24 h. Reactions were quenched by ultrafiltration using 10 kDa molecular weight cutoff (MWCO) centrifugal filters (VWR International GmbH, Darmstadt, Germany). Filtrates were analyzed by RP‐HPLC and ESI‐MS (Agilent GmbH, Waldbronn, Germany) to quantify hydrolysis products and identify cleavage sites. Control reactions lacking the enzyme were processed identically and showed no detectable substrate conversion over 24 h (see SI, Figure S39).

**Bulk polyamide assays.** Hydrolysis of bulk polyamides was performed under the same buffer and temperature conditions used for the oligomer assays. Nylon‐6 film (0.2 mm thickness; Goodfellow GmbH, Hamburg, Germany) was cut into approximately 3 × 3 mm pieces (≈2 mg) and incubated with 1 µM NylC‐HP, corresponding to 0.67 wt% substrate and an enzyme‐to‐substrate ratio of 0.5 mM g^−1^. Nylon‐6,6 granule (3 mm diameter; Goodfellow GmbH, Hamburg, Germany) assays were performed with one granule (≈11 mg) and 3.2 µM NylC‐HP (2.16 wt%, enzyme‐to‐substrate ratio 0.3 mM g^−1^). All reactions were incubated for 48 h with shaking. Supernatants were collected by ultrafiltration and analyzed by RP‐HPLC and ESI‐MS to determine soluble hydrolysis products.

## Conflicts of Interest

The authors declare no conflicts of interest.

## Supporting information

Supplementary Material
