# Supplementary material for: Defined Nylon Oligomers Enable Mechanistic Insight Into Enzymatic Polyamide Depolymerization
Source: ChemSusChem. 2026 Jun 9;19(11):e70693. doi: 10.1002/cssc.70693 (PMC13248228; doi:10.1002/cssc.70693)
Supplement: Supplementary file 1 — Supplementary Material [file CSSC-19-e70693-s001.pdf]

Supporting Information

Defined Nylon Oligomers Enable Mechanistic Insight into Enzymatic Polyamide Depolymerization

Sukmanita Dewi[1], Hendrik Puetz[2], Ulrich Schwaneberg[2], Laura Hartmann[1,3*]

[1]Institute of Macromolecular Chemistry, Albert-Ludwigs-University of Freiburg, Freiburg, Germany

[2]Institute of Biotechnology, RWTH Aachen University, Aachen, Germany

[3]Freiburg Materials Research Center, Albert-Ludwigs-University of Freiburg, Freiburg, Germany

Materials

All reagents were used without further purification.

Solvents and General Chemicals

Dichloromethane (DCM, HPLC grade), dimethylformamide (DMF, peptide synthesis grade), ethyl acetate (analytical grade), methanol (MeOH, HPLC grade), toluene (analytical grade), n-hexane (analytical grade), piperidine (99%), pyridine (≥99%), triethylamine (≥99%), trifluoroacetic acid (TFA, 99%), triethylsilane (TES, 98%), 1,6-hexanediamine (≥99%), 1,1,1,3,3,3-hexafluoro-2-propanol (HFIP, ≥99%), dipotassium hydrogen phosphate (K_2HPO_4, ≥99%), potassium dihydrogen phosphate (KH_2PO_4, ≥99%), and sodium hydroxide (99%) were purchased from Fisher Scientific. 9-Fluorenylmethoxycarbonyl chloride (Fmoc-Cl, 98%) and triphenylmethyl chloride (Trt-Cl, 98%) were purchased from BLD Pharmatech GmbH. Diethyl ether (analytical grade), tetrahydrofuran (THF, analytical grade), citric acid (99%), magnesium sulfate ($MgSO_4$, 99%), sodium chloride (≥99%), sodium bicarbonate ($NaHCO_3$, ≥99%), and potassium carbonate (K_2CO_3, ≥99%) were purchased from Carl Roth GmbH. Chloroform-d (≥99%), DMSO-d_6 (≥99%), and formic acid-d_2 were obtained from Deutero GmbH. Acetonitrile (HPLC grade), acetic anhydride (99%), adipic acid (≥99%), di-tert-butyl dicarbonate (Boc_2O, 99%), hydrochloric acid (≥99%), magnesium chloride (≥98%), and triisopropylsilane (TIPS, 98%) were purchased from Sigma-Aldrich. Ultrapure water (18.0 MΩ·cm resistivity) was produced using a Milli-Q system (Elga GmbH, Vienna, Austria).

Coupling and Assay Reagents

Benzotriazol-1-yl-oxytripyrrolidinophosphonium hexafluorophosphate (PyBOP, 98%) was purchased from Apollo Scientific. Fmoc-6-amino hexanoic acid (Fmoc-PA6, 99%) and 2,4,6-mesitylenesulfonyl-3-nitro-1,2,4-triazole (MSNT, 97%) were purchased from BLD Pharmatech GmbH. *N,N'*-diisopropylcarbodiimide (DIC, 99%), diisopropylethylamine (DIPEA, ≥99%), and 1-hydroxybenzotriazole (HOBt, 97%) were purchased from Sigma-Aldrich. 4-(*N,N*-dimethylamino)pyridine (DMAP, 99%), 1-methylimidazole (MeIm, 99%), and Bradford assay kit (Pierce Coomassie Plus) were purchased from Fisher Scientific. Centrifugal ultrafiltration units (MWCO 10 kDa) were purchased from VWR BDH Chemicals. PHB-Wang resin (loading 0.97 mmol g^{-1}) was purchased from Rapp Polymere. Resin handling was carried out in 10 ml fritted polypropylene syringe reactors from Multisyntech GmbH.

Monomers and Nylon Standards

ε-Caprolactam (≥99%) and 6-aminohexanoic acid (6-AHA, ≥99%) were purchased from Fisher Scientific. Nylon-6 films (0.2 mm thickness, 4.3% crystallinity by DSC) and nylon-6,6 granules (3 mm diameter, 8.3% crystallinity by DSC) were purchased from Goodfellow GmbH.

Instrumentation

Nuclear Magnetic Resonance Spectroscopy (NMR)

^{1}H-NMR and ^{13}C-NMR spectra were recorded on a Bruker AVANCE Neo 400 spectrometer at room temperature. Chemical shifts (δ) are reported in parts per million (ppm) and are referenced to the residual solvent signals ($CDCl_3$: δ ^{1}H 7.26 ppm, DMSO-d_6: δ ^{1}H 2.50 ppm, ^{13}C 39.52 ppm). Coupling constants (J) are reported in Hertz (Hz). Multiplicity abbreviations are as follows: s = singlet, d = doublet, t = triplet, q = quartet, m = multiplet, br = broad.

Reversed Phase – High Pressure Liquid Chromatography – Mass Spectrometry (RP-HPLC-MS)

RP-HPLC-MS analysis was performed on an Agilent Technologies 6120 series system equipped with a binary pump, a C18 reversed-phase column (3.0 mm × 50 mm, 3 µm particle size; MZ- Aqua Perfect C18, Agilent Technologies, Waldbronn, Germany), a quadruple mass spectrometer (ESI-MS) operating in the m/z 200–2000 range, and a UV/Vis detector. Mobile phase A consisted of water with 0.1% (v/v) formic acid, and mobile phase B consisted of 100% acetonitrile. All analyses were carried out at a flow rate of 0.4 mL min^{-1}. The gradient programs used for the different analytical applications are summarized in Table S1. UV and MS data were processed using OpenLab ChemStation software (Agilent Technologies).

Table S1. RP-HPLC-MS gradient programs and detection parameters.

Application	Injection volume	Method	UV detector
Synthesis of building block	1 µL	5-95% B over 15 min	214 nm
Synthesis of nylon oligomers	1 µL	1-50% B over 15 min	205 nm
Oligomer + Bulk PA66 assays	5 µL	100% A for 3 min → 0-50% B over 15 min	205 nm
Bulk PA6 assays	5 µL	100% A for 5 min → 0-10% B over15 min	205 nm

Preparative Reversed Phase – High Pressure Liquid Chromatography (Prep-RP-HPLC)

Preparative RP-HPLC was performed on an Agilent Technologies 1260 Infinity system equipped with a variable wavelength detector (VWD) set to 205 nm. A Pursuit 10 C18 column (250 x 10 mm, 10 µm; Agilent Technologies, Waldbronn, Germany) was used for all purifications. The mobile phase A consisted of water containing 0.1% (v/v) formic acid, and mobile phase B consisted of acetonitrile containing 0.1% (v/v) formic acid. Samples were purified at 25 °C and a flow rate of 10 mL min^{-1} using a linear gradient from 15% B to 35% B over 18 min for PA6 hexamer ($PA6_6$), PA6 heptamer ($PA6_7$), and PA66 tetramer ($PA66_4$). Product-containing fractions were collected using an automated fraction collector, combined, concentrated, and lyophilized.

Electrospray Ionization Mass Spectrometry (ESI-MS)

High-resolution mass spectra were recorded on an Agilent 6546 LC/Q-TOF mass spectrometer (Agilent Technologies, Waldbronn, Germany) equipped with an electrospray ionization (ESI) source operating in positive ion mode over an *m/z* range of 50–3200. ESI-MS analysis was carried out only for the highest molecular weight oligomers ($PA6_7$ and $PA66_4$), which were dissolved in HFIP to ensure complete dissolution. Data acquisition and processing were carried out using Agilent MassHunter software.

Flash Column Chromatography

Flash column chromatography was performed on an Interchim PuriFlash 5 system equipped with a UV scan detector (200-400 nm) using silica columns (Puriflash F0040, 30 µm; Interchim Deutschland GmbH, Mannheim, Germany). The Fmoc-PA66 building block was purified using a solvent gradient of DCM and MeOH, starting from 100% DCM and gradually increasing to a 10:1 (v/v) DCM/MeOH mixture. Fractions containing the desired product were collected and concentrated under reduced pressure.

Freeze Dryer

Lyophilization of the final oligomers was carried out on an Alpha 3–4 LSCbasic freeze dryer (Martin Christ Freeze Dryers GmbH, Osterode am Harz, Germany). Samples were dried at −99 °C and 0.1 mbar until complete removal of solvent.

Centrifuges

Ultrafiltration of enzymatic assay samples was performed in a CF-10 High-Performance centrifuge (Witeg GmbH, Wertheim, Germany) at 13,500 rpm for 4 min.

Synthesis and Characterization of the Building Block Fmoc-PA66

The compound 6-((6-((((9*H*-fluoren-9-yl)methoxy)carbonyl)amino)hexyl)amino)-6-oxohexanoic acid (**Fmoc-PA66**) was prepared following a four-step procedure adapted from previously published general methods for the synthesis of Fmoc-protected building blocks EDS and ODS.[1–3] Adipic anhydride (**4**) was synthesized according to the general literature procedure,[4] with minor modifications, because commercially available material contained impurities that interfered with the final coupling step. The overall isolated yield of the synthesis was 49%.

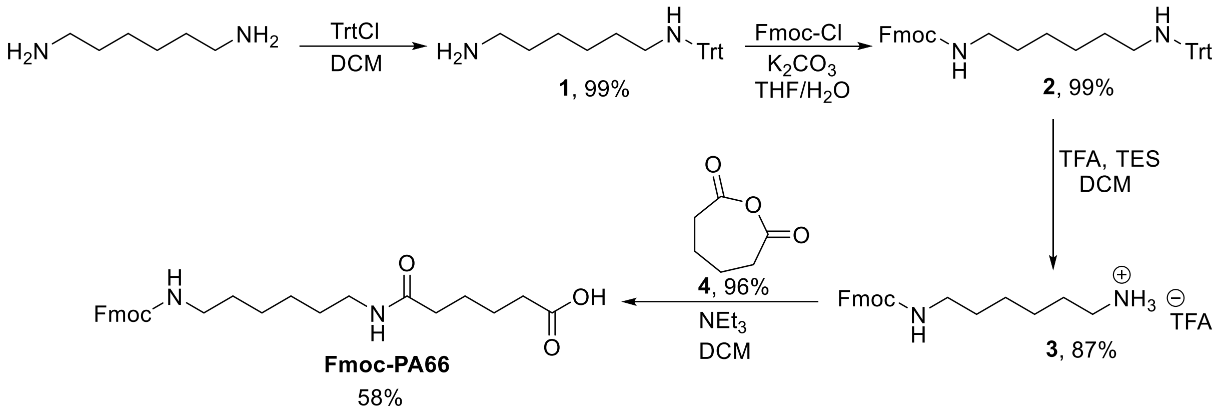

Scheme S1. Synthetic route to the Fmoc-protected nylon-6,6 building block (**Fmoc-PA66**) for solid-phase assembly.

N^1-tritylhexane-1,6-diamine (1)

In a round-bottom flask, 1,6-Hexanediamine (23.2 g, 200 mmol, 1.0 equiv) was dissolved in DCM (250 mL) under an inert atmosphere and the solution was cooled to 0 °C. A solution of trityl chloride (13.9 g, 50.0 mmol, 0.25 equiv) in DCM (75 mL) was added dropwise over 1 h. The resulting white slurry mixture was stirred overnight while warming to room temperature. The reaction mixture was concentrated to half of its volume under reduced pressure and washed with saturated aqueous $NaHCO_3$ solution (3 x 100 mL). The combined organic layers were dried over $MgSO_4$, filtered, and the solvent was removed under reduced pressure to give **1** as a pale yellow oil (17.8 g, 49.6 mmol, 99%). The product was used directly in the next step without further purification. ^{1}H-NMR (400 MHz, $CDCl_3$): δ (ppm) = 7.50 – 7.41 (m, 6H, H-2), 7.30 – 7.22 (m, 6H, H-1), 7.21 – 7.11 (m, 3H, H-3), 2.70 (t, 2H, H-8), 2.27 (br s, 3H, H-4), 2.09 (t, 2H, H-5), 1.51 – 1.41 (m, 4H, H-6), 1.34 – 1.21 (m, 4H, H-7).

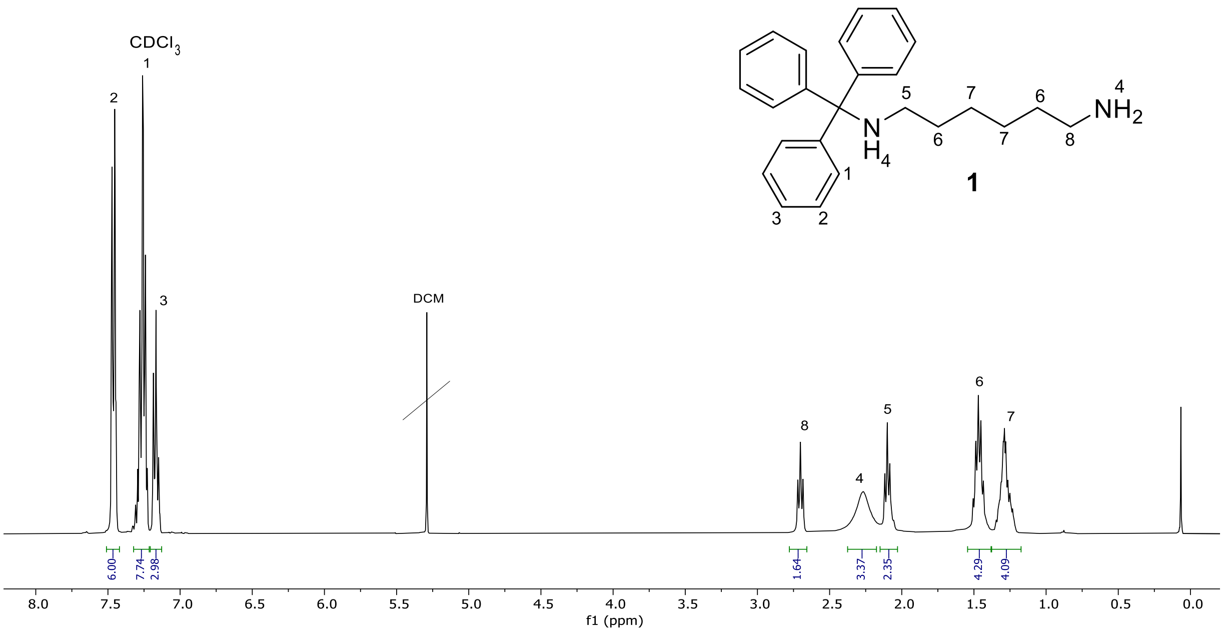

Figure S1. ^{1}H-NMR spectrum of **1** (400 MHz, $CDCl_3$).

(9*H*-Fluoren-9-yl)methyl (6-(tritylamino)hexyl)carbamate (2)

Compound **1** (17.8 g, 49.6 mmol, 1.0 equiv) was dissolved in THF (150 mL), and a solution of K_2CO_3 (34.3 g, 248 mmol, 5.0 equiv) in water (150 mL) was added. Fmoc-Cl (13.5 g, 52.1 mmol, 1.05 equiv) was then added, and the biphasic mixture was stirred vigorously overnight. THF was removed under reduced pressure, and the remaining yellow oil on the aqueous phase was extracted with ethyl acetate (200 mL). The organic layer was washed with water (3 x 100 mL), dried over $MgSO_4$, filtered, and concentrated under reduced pressure to afford **2** as a yellow oil (26.6 g, 49.1 mmol, 99%). The product was used directly in the next step without further purification. ^{1}H-NMR (400 MHz, $CDCl_3$): δ (ppm) = 7.80 – 7.75 (m, 2H, H-14), 7.60 (dd, 2H, H-11), 7.51 – 7.44 (m, 6H, H-2), 7.44 – 7.36 (m, 2H, H-13), 7.35 – 7.23 (m, 8H, H-1; H-12), 7.21 – 7.13 (m, 3H, H-3), 4.69 (br s, 1H, H-4), 4.40 (d, 2H, H-9), 4.21 (t, 1H, H-10), 3.18 – 3.13 (m, 2H, H-8), 2.12 (t, 2H, H-5), 1.51 – 1.41 (m, 4H, H-6), 1.34 – 1.21 (m, 4H, H-7).

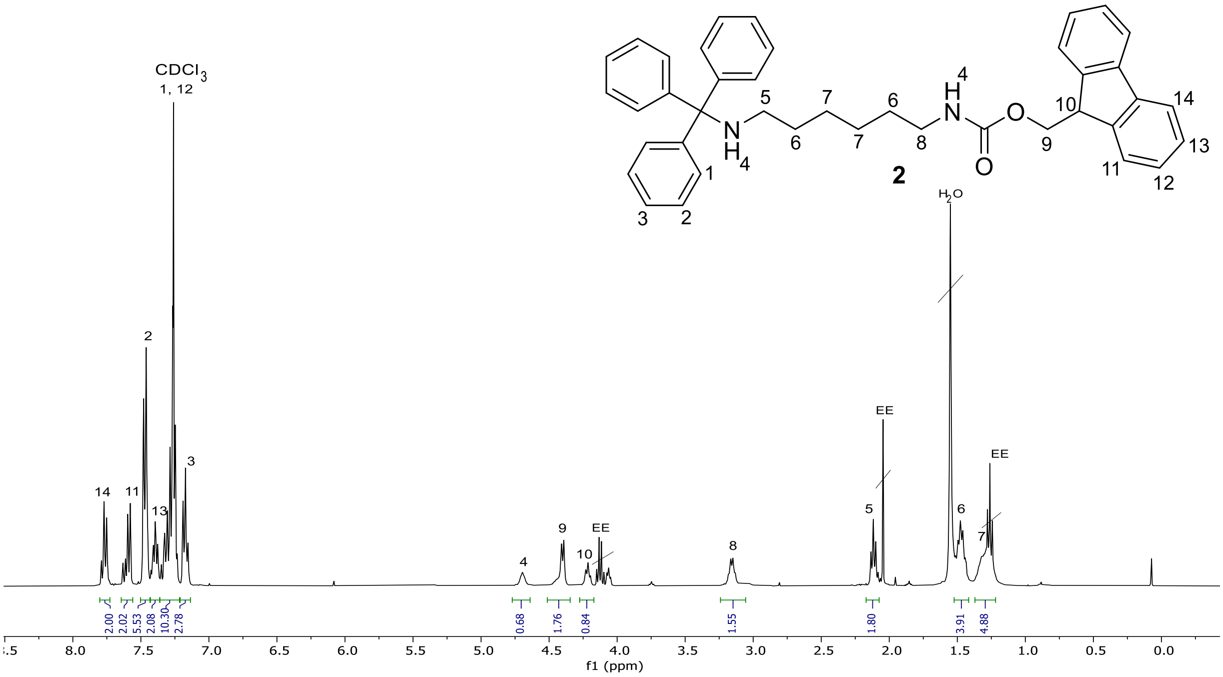

Figure S2. ^{1}H-NMR spectrum of **2** (400 MHz, $CDCl_3$).

6-((((9*H*-Fluoren-9-yl)methoxy)carbonyl)amino)hexan-1-aminium trifluoroacetate (3)

To a solution of **2** (26.6 g, 49.1 mmol, 1.0 equiv) in DCM (350 mL), TES (15.7 mL, 98.2 mmol, 2.0 equiv) and TFA (18.8 mL, 245 mmol, 5.0 equiv) were added slowly. The reaction mixture was stirred at room temperature for 1 h, forming a turbid pale yellow solution. After removal of DCM under reduced pressure, residual TFA was co-evaporated with toluene (4 x 50 mL). The crude product was dissolved in DCM (50 mL) and precipitated into diethyl ether (400 mL). The resulting solid was filtered, washed with diethyl ether, and dried under high vacuum to afford the TFA salt of **3** as a white powder (19.4 g, 42.8 mmol, 87%) in high purity (>99% as determined by RP-HPLC analysis). ^{1}H-NMR (400 MHz, $CDCl_3$): δ (ppm) = 7.97 (br s, 3H, H-13), 7.74 (d, 2H, H-4), 7.56 (d, 2H, H-1), 7.39 – 7.35 (m, 2H, H-3), 7.33 – 7.24 (m, 2H, H-2), 4.92 (br s, 1H, H-7), 4.35 (d, 2H, H-6), 4.17 (t, 1H, H-5), 3.13 – 3.07 (m, 2H, H-8), 2.92 – 2.86 (m, 2H, H-12), 1.67 – 1.60 (m, 2H, H-11), 1.47 – 1.40 (m, 2H, H-9), 1.39 – 1.17 (m, 4H, H-10). ESI-MS: *m/z* calcd. for $C_{21}H_{27}N_2O_2$ [M]$^+$ 339.2; found 339.2.

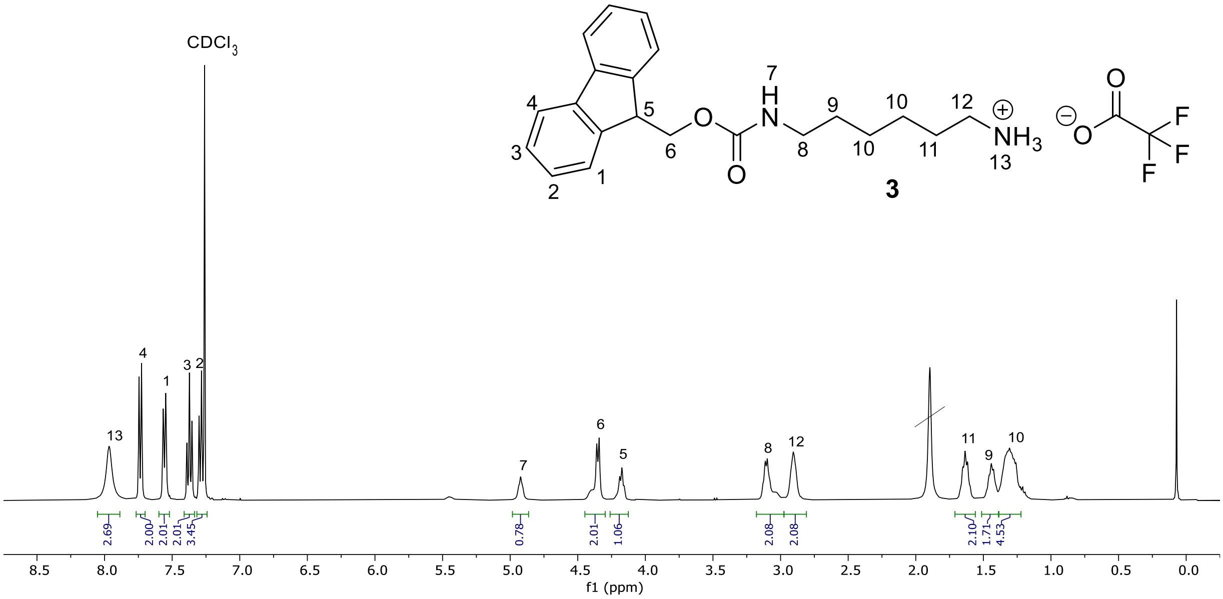

Figure S3. ^{1}H-NMR spectrum of **3** (400 MHz, $CDCl_3$).

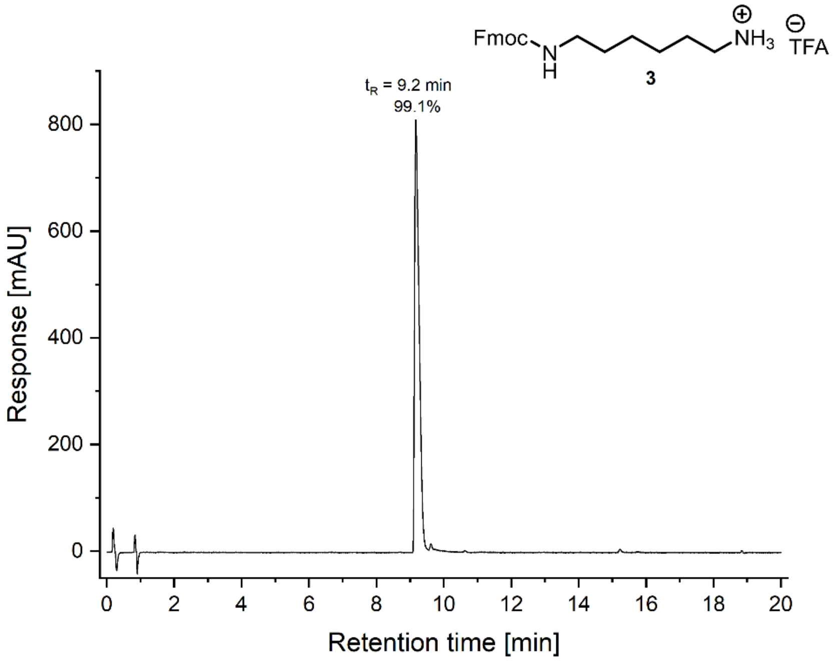

Figure S4. RP-HPLC chromatogram of **3** (linear gradient from 5-95% acetonitrile over 15 min; UV detection at 214 nm).

Adipic anhydride (4)

Adipic acid (12.0 g, 82.1 mmol, 1.0 equiv), Boc_2O (17.9 g, 82.1 mmol, 1.0 equiv), and magnesium chloride (0.2 g, 1.6 mmol, 0.02 equiv) were dissolved in THF (30 mL) and stirred at 40 °C for 1 h, or until gas evolution has ceased. A white precipitate formed during the reaction. The solid was collected by filtration, washed thoroughly with THF and cold n-hexane, and dried under high vacuum to give **4** as a white powder (10.1 g, 78.5 mmol, 96%). ^{1}H-NMR (400 MHz, $CDCl_3$): δ (ppm) = 2.56 – 2.47 (m, 4H, H-1), 1.79 – 1.72 (m, 4H, H-2).

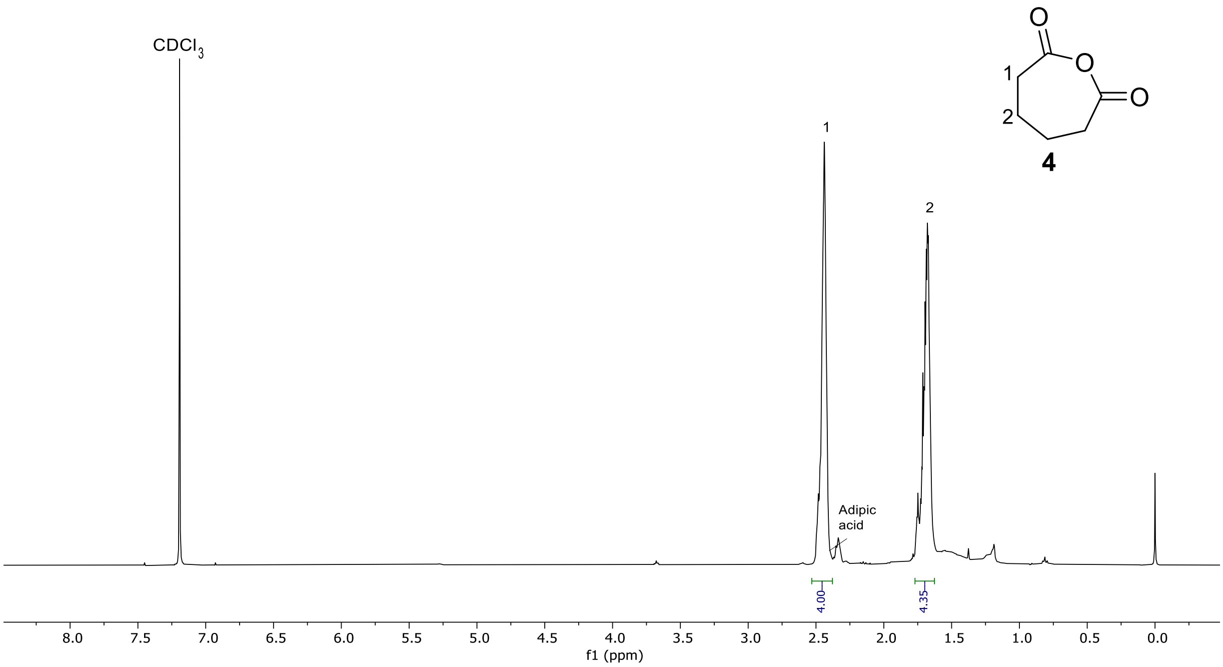

Figure S5. ^{1}H-NMR spectrum of **4** (400 MHz, $CDCl_3$).

6-((6-((((9*H*-Fluoren-9-yl)methoxy)carbonyl)amino)hexyl)amino)-6-oxohexanoic acid (Fmoc-PA66)

Compound **3** (3.0 g, 6.6 mmol, 1 equiv) was suspended in DCM (100 mL), and triethylamine (1.9 mL, 13.9 mmol, 2.1 equiv) was added to dissolve the material. This solution was added dropwise over 1 h to a cooled (0 °C) solution of **4** (1.3 g, 9.9 mmol, 1.5 equiv) in DCM (175 mL). The mixture was stirred for an additional 15 min after the dropwise is finished, during which a white precipitate formed. The solid was removed by filtration, and the filtrate was washed with 30% aqueous citric acid (3 x 100 mL). The organic layer was dried over $MgSO_4$, filtered, and concentrated under reduced pressure. The crude product was then dissolved in a small volume of DMF and precipitated into 2.5 M HCl, yielding a white solid. The precipitate was collected by filtration and dried under high vacuum to afford **Fmoc-PA66** as a white powder (1.8 g, 3.8 mmol, 58%) with a purity of >80% (RP-HPLC). The overall yield of the synthesis was 49%. For analytical purposes, a portion of the material was further purified by flash column chromatography to obtain a reference sample of >99% purity (Figure S8). All subsequent reactions in this work were performed using the crude monomer. ^{1}H-NMR (400 MHz, DMSO-d_6): δ (ppm) = 7.88 (d, 2H, H-4), 7.73 (t, 1H, H-11), 7.68 (d, 2H, H-1), 7.45 – 7.38 (m, 2H, H-3), 7.35 – 7.29 (m, 2H, H-2), 7.24 (t, 1H, H-7), 4.29 (d, 2H, H-6), 4.20 (t, 1H, H-5), 3.05 – 2.91 (m, 4H, H-8), 2.24 – 2.15 (m, 2H, H-14), 2.08 – 2.00 (m, 2H, H-12), 1.52 – 1.43 (m, 4H, H-13), 1.42 – 1.31 (m, 4H, H-9), 1.27 – 1.17 (m, 4H, H-10). ^{13}C-NMR (400 MHz, DMSO-d_6): δ (ppm) = 171.63 (C-14, C-19), 156.05 (C-9), 143.93 (C-1), 140.72 (C-6), 127.56 (C-4), 127.00 (C-5), 125.11 (C-3), 120.08 (C-2), 65.1 (C-8), 46.78 (C-7), 38.31 (C-

10), 35.12 (C-13), 29.3 (C-15), 29.13 (C-18), 26.1 (C-11), 25.91 (C-16), 24.88 (C-12), 24.18 (C-17).ESI-MS: *m/z* calcd. for $C_{27}H_{34}N_2O_5$ $[M+H]^+$ 467.2; found 467.2.

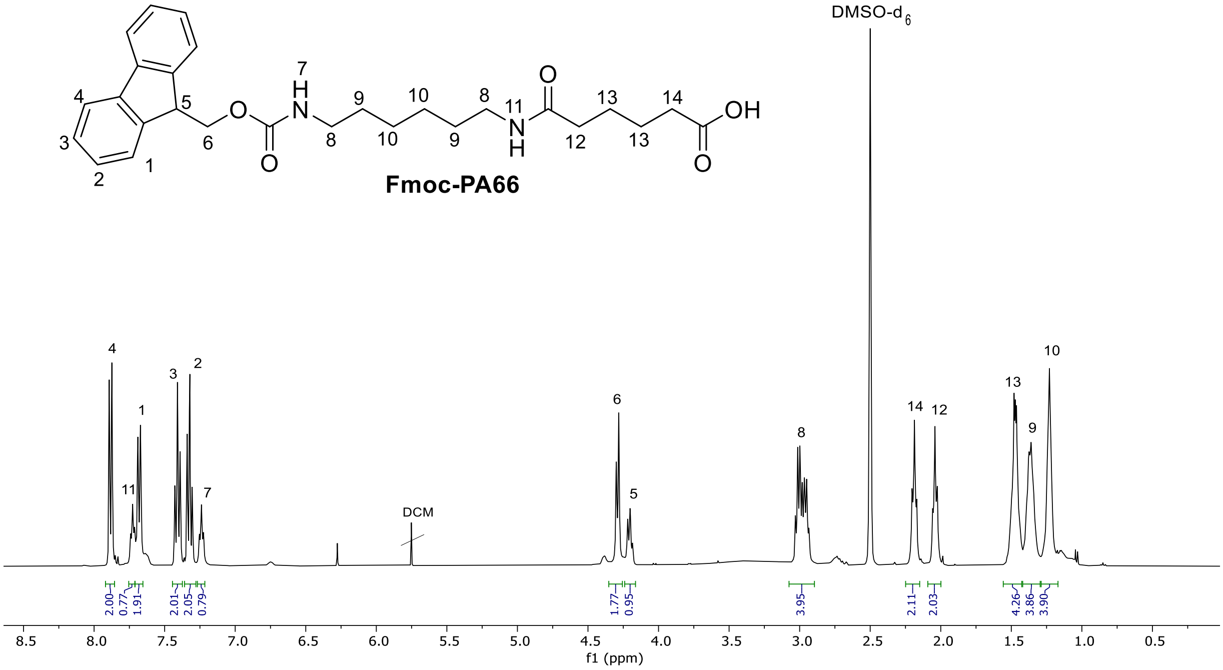

Figure S6. ^{1}H-NMR spectrum of **Fmoc-PA66** (400 MHz, DMSO-d$_6$).

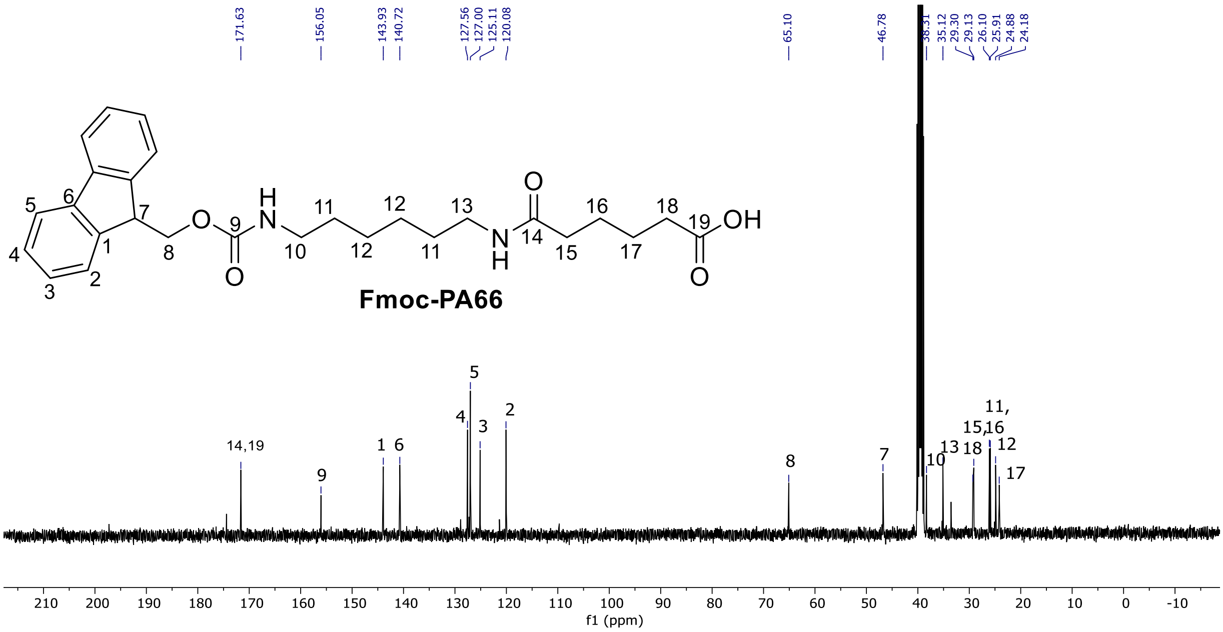

Figure S7. ^{13}C-NMR spectrum of **Fmoc-PA66** (400 MHz, DMSO-d$_6$). Unassigned signals are marked with an asterisk (*).

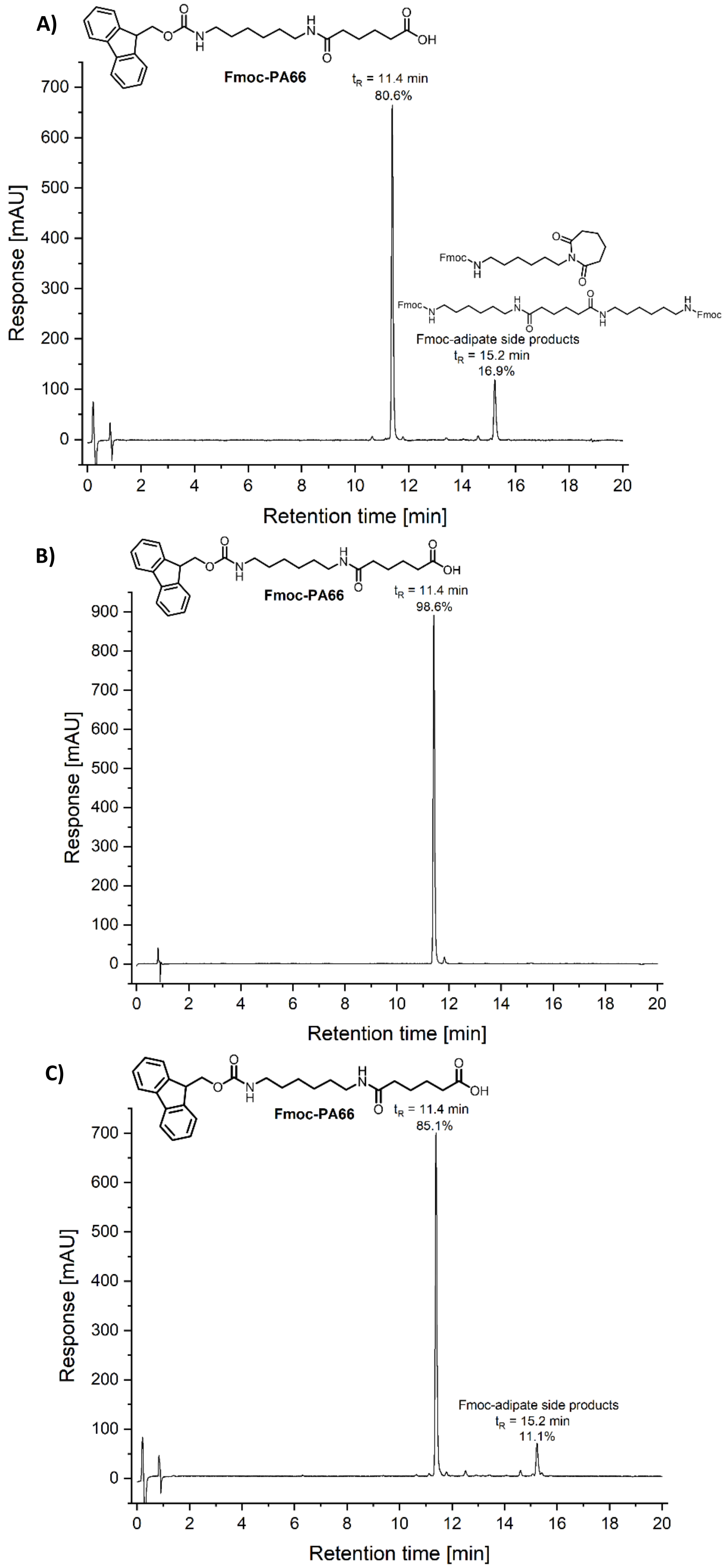

Figure S8. RP-HPLC chromatograms of **Fmoc-PA66**: A) unpurified crude product, B) monomer purified by flash column chromatography, and C) monomer recovered after solid-phase synthesis by precipitation in 2.5 M HCl. Chromatograms were recorded using a linear gradient from 5-95% acetonitrile over 15 min with UV detection at 214 nm.

Solid Phase Synthesis of Nylon Oligomers

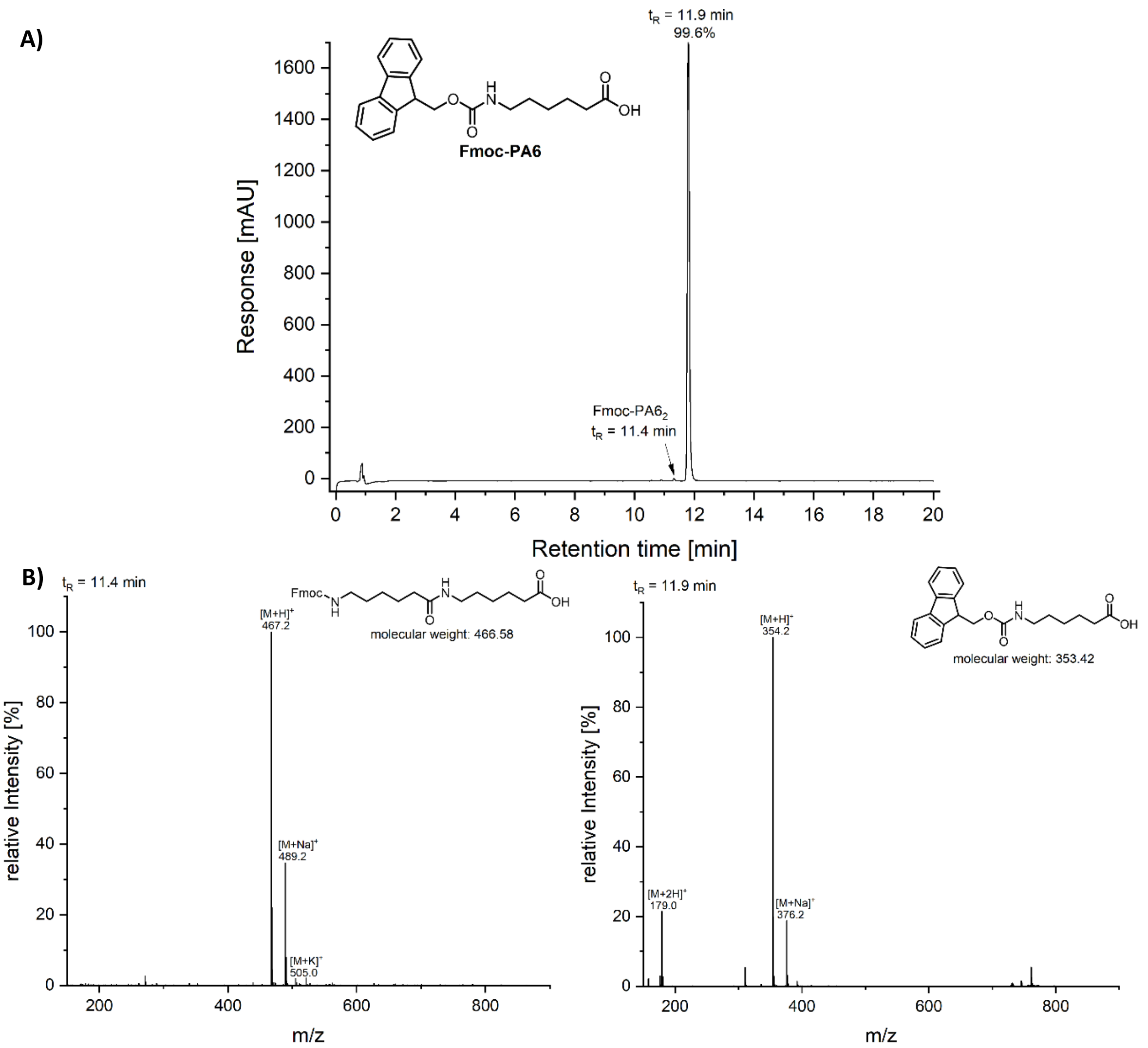

Figure S9. Analytical characterization of commercial Fmoc-PA6 by RP-HPLC and ESI-MS. A) RP-HPLC chromatogram recorded using a linear gradient from 5-95% acetonitrile over 15 min with UV detection at 214 nm. B) Corresponding ESI-MS spectra of the detected peaks.

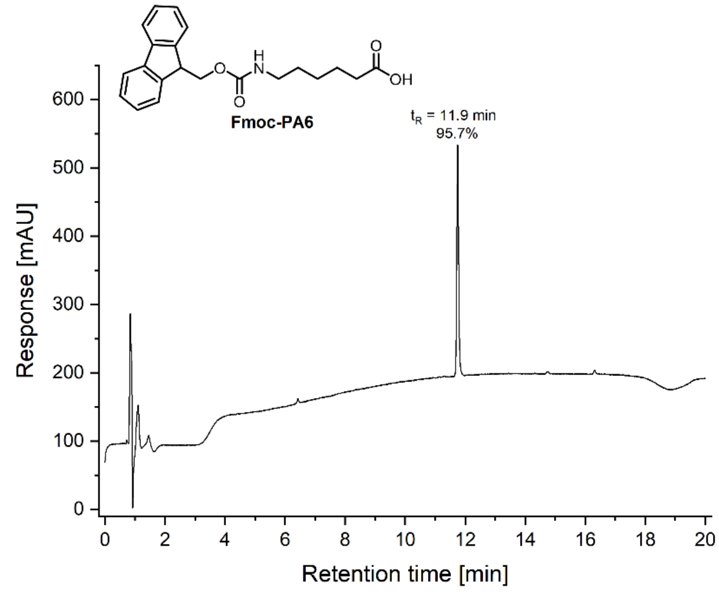

Figure S10. RP-HPLC of recovered Fmoc-PA6 monomer after precipitation in 2.5 M HCl solution. Chromatograms were recorded using a linear gradient from 5-95% acetonitrile over 15 min with UV detection at 214 nm.

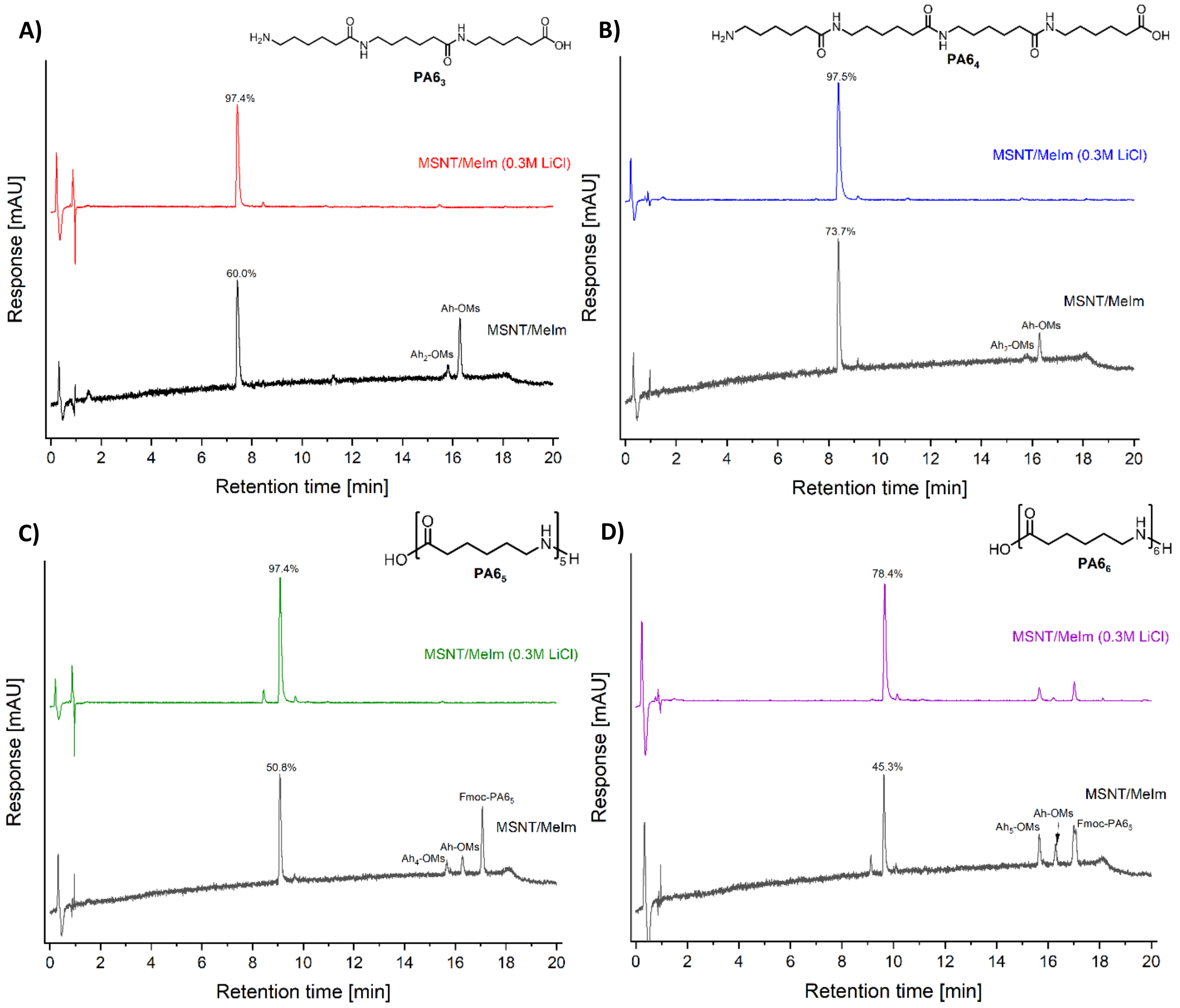

Figure S11. Effect of LiCl on product purity during the solid-phase synthesis of PA6 oligomers using MSNT/MeIm activation. RP-HPLC chromatograms of **A)** trimer ($PA6_3$), **B)** tetramer ($PA6_4$), **C)** pentamer ($PA6_5$), and **D)** hexamer ($PA6_6$) synthesized with and without 0.3 M LiCl in DMF. Chromatograms were recorded using a linear gradient from 1-50% acetonitrile over 15 min with UV detection at 205 nm. Oligomer purities were determined by peak integration.

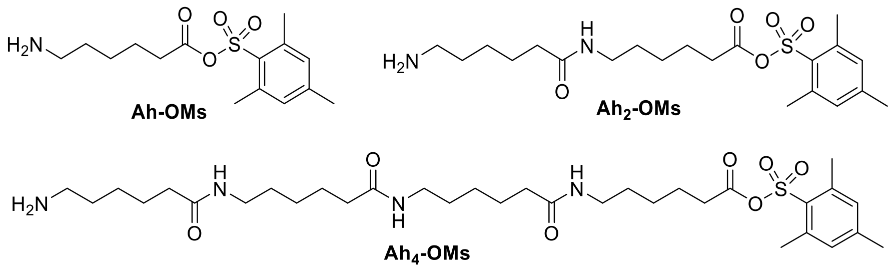

Figure S12. Representative reactive side products formed during MSNT-mediated activation: mesitylenesulfonyl-activated oligomers (Ah_x-OMs).

Nylon-6 Dimer ($PA6_2$)

^{1}H-NMR (400 MHz, DMSO-d_6): δ (ppm) = 11.99 (br s, 1H, H-10), 7.74 (t, 1H, H-7), 7.66 (br s, 2H, H-1), 3.00 (q, 2H, H-8), 2.80 – 2.70 (m, 2H, H-2), 2.18 (t, 2H, H-9), 2.04 (t, 2H, H-6), 1.57 – 1.41 (m, 6H, H-3), 1.36 (p, 2H, H-5), 1.30 – 1.13 (m, 4H, H-4). ESI-MS: *m/z* calcd. for $C_{12}H_{24}N_2O_3$ $[M+H]^+$ 245.2; found 245.2.

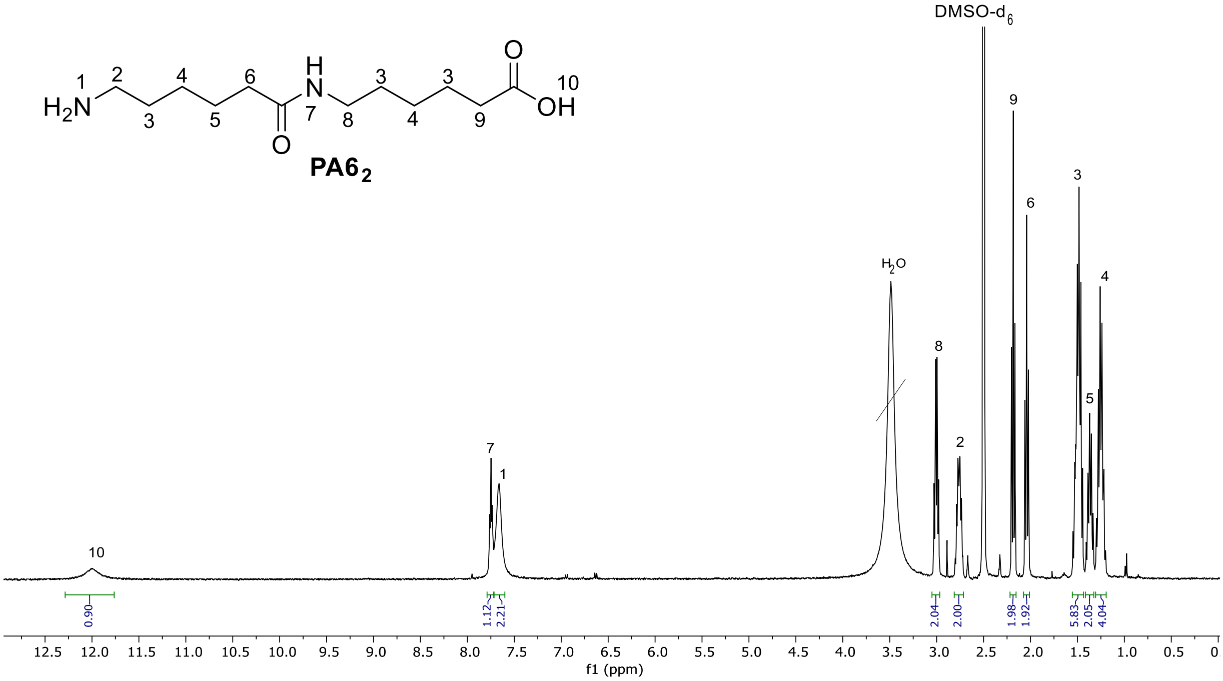

Figure S13. ^{1}H-NMR spectrum of **$PA6_2$** (400 MHz, DMSO-d_6).

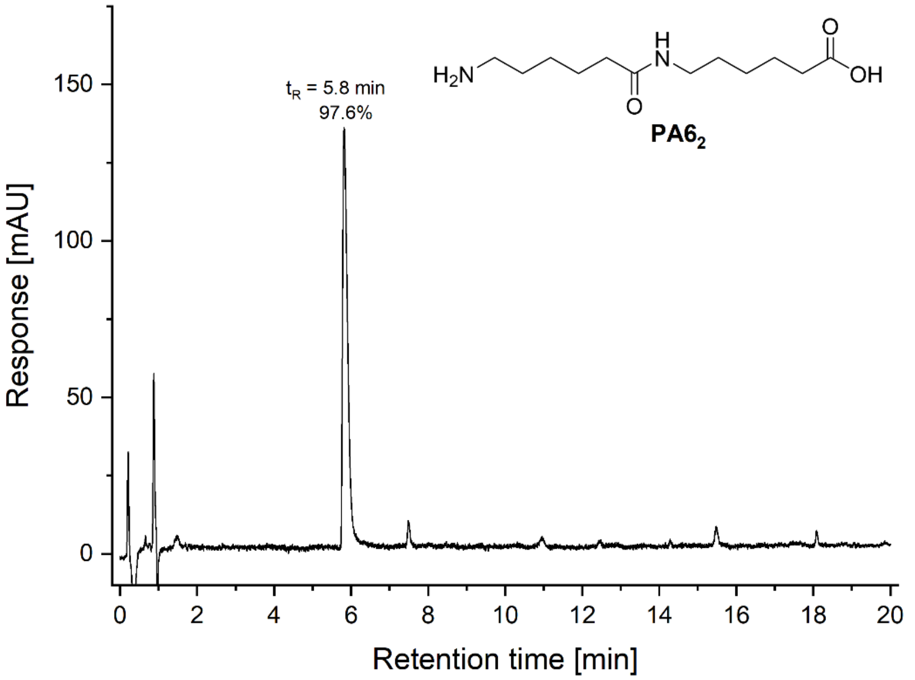

Figure S14. RP-HPLC chromatogram of **$PA6_2$** (linear gradient from 1-50% acetonitrile over 15 min; UV detection at 205 nm).

Nylon-6 Trimer ($PA6_3$)

^{1}H-NMR (400 MHz, DMSO-d_6): δ (ppm) = 11.99 (br s, 1H, H-10), 7.72 (q, 2H, H-7), 7.62 (br s, 2H, H-1), 2.99 (q, 4H, H-8), 2.80 – 2.70 (m, 2H, H-2), 2.17 (t, 2H, H-9), 2.02 (q, 4H, H-6), 1.57 – 1.41 (m, 8H, H-3), 1.36 (p, 4H, H-5), 1.30 – 1.13 (m, 6H, H-4). ESI-MS: *m/z* calcd. for $C_{18}H_{35}N_3O_4$ $[M+H]^+$ 358.3; found 358.2.

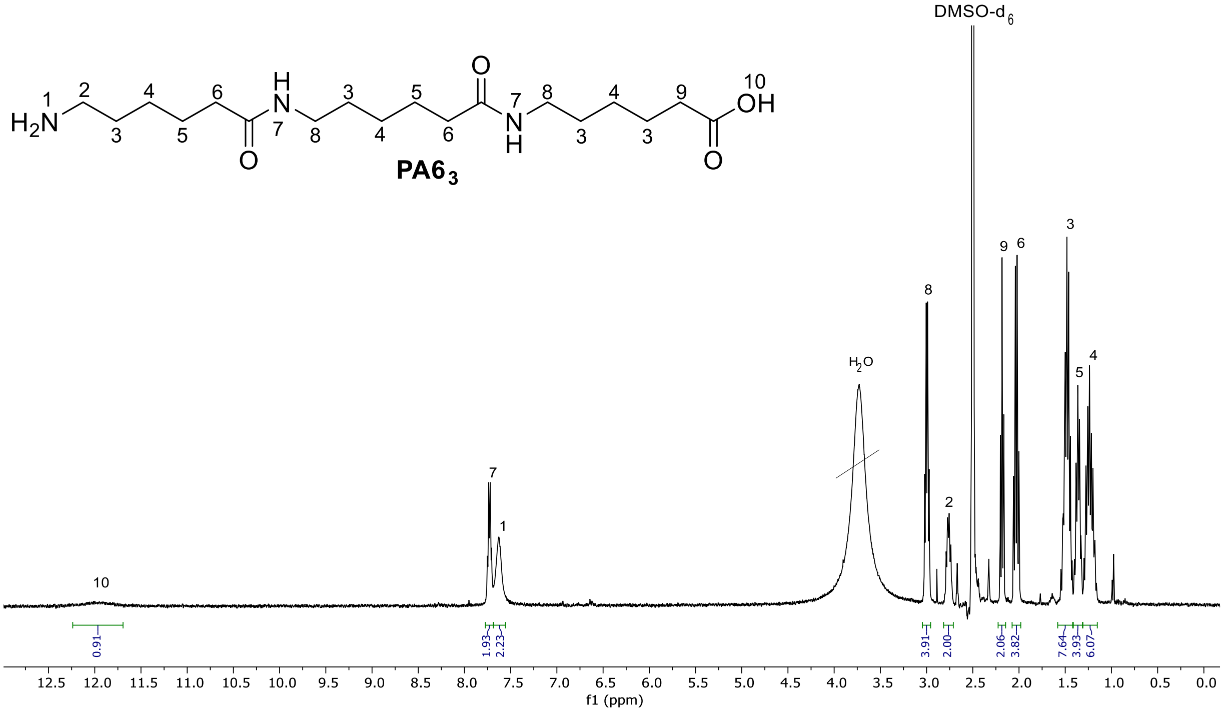

Figure S15. ^{1}H-NMR spectrum of **PA6$_3$** (400 MHz, DMSO-d_6).

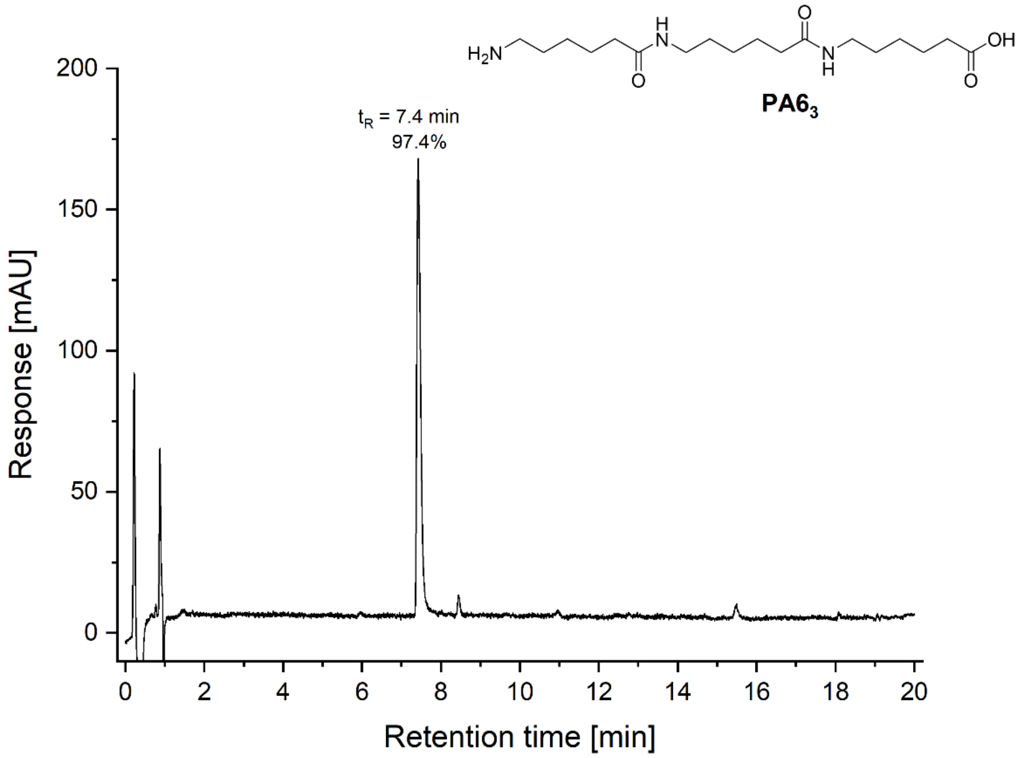

Figure S16. RP-HPLC chromatogram of **PA6$_3$** (linear gradient from 1-50% acetonitrile over 15 min; UV detection at 205 nm).

Nylon-6 Tetramer (PA6_4)

^{1}H-NMR (400 MHz, DMSO-d_6): δ (ppm) = 11.98 (br s, 1H, H-10), 7.73 (p, 3H, H-7), 7.65 (br s, 2H, H-1), 2.99 (q, 6H, H-8), 2.80 – 2.70 (m, 2H, H-2), 2.18 (t, 2H, H-9), 2.06 – 1.99 (m, 6H, H-6), 1.57 – 1.41 (m, 10H, H-3), 1.40 – 1.30 (m, 6H, H-5), 1.30 – 1.13 (m, 8H, H-4). ESI-MS: *m/z* calcd. for $C_{24}H_{46}N_4O_5$ $[M+H]^+$ 471.4; found 471.4.

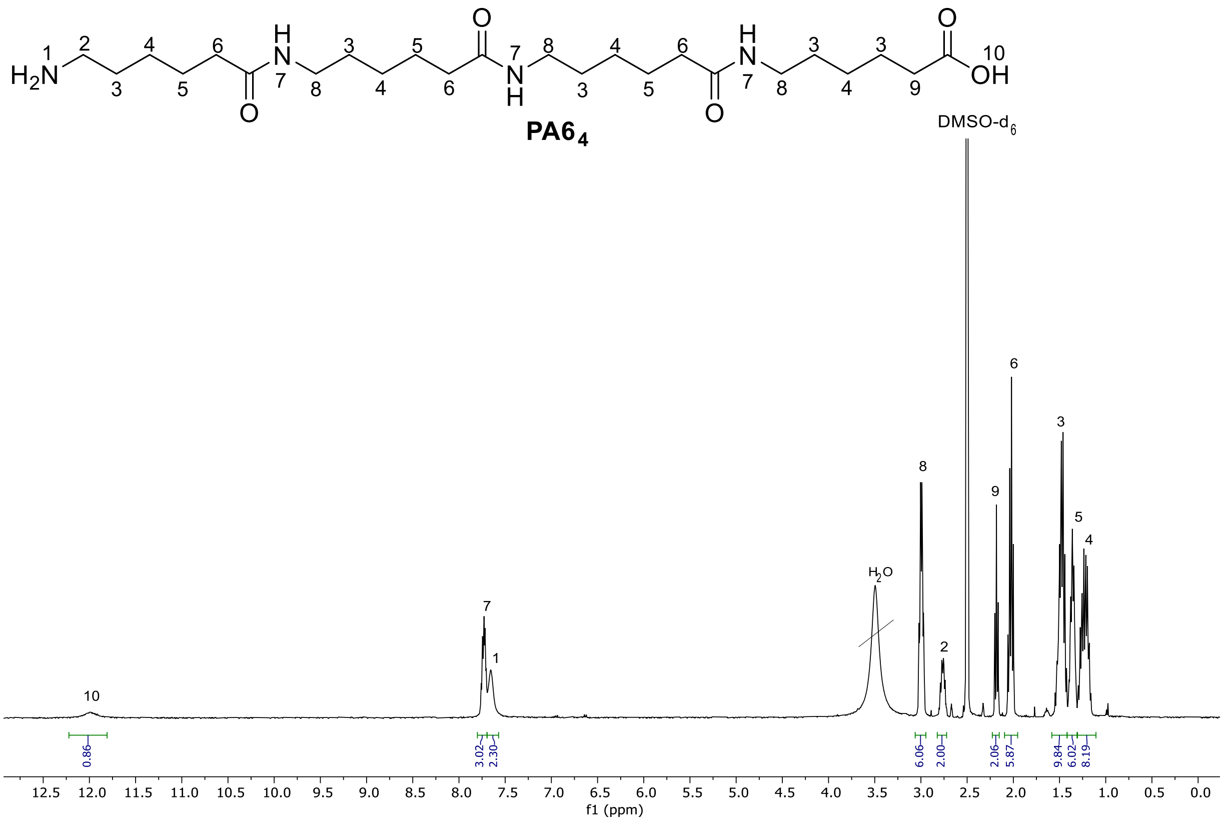

Figure S17. ^{1}H-NMR spectrum of **PA6_4** (400 MHz, DMSO-d_6).

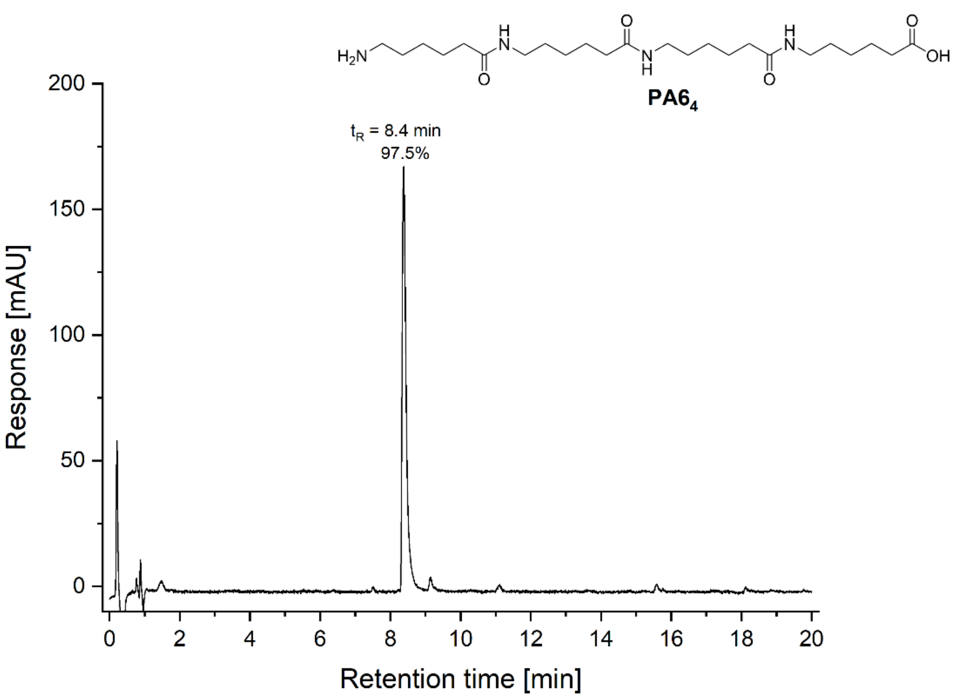

Figure S18. RP-HPLC chromatogram of **PA6_4** (linear gradient from 1-50% acetonitrile over 15 min; UV detection at 205 nm).

Nylon-6 Pentamer (PA6$_5$)

^{1}H-NMR (400 MHz, DMSO-d$_6$): δ (ppm) = 11.98 (br s, 1H, H-10), 7.72 (dt, 4H, H-7), 7.62 (br s, 2H, H-1), 2.99 (dq, 8H, H-8), 2.82 – 2.70 (m, 2H, H-2), 2.17 (t, 2H, H-9), 2.07 – 1.97 (m, 8H, H-6), 1.57 – 1.41 (m, 12H, H-3), 1.40 – 1.30 (m, 8H, H-5), 1.30 – 1.13 (m, 10H, H-4). ESI-MS: *m/z* calcd. for $C_{30}H_{57}N_5O_6$ $[M+H]^+$ 584.4; found 584.4.

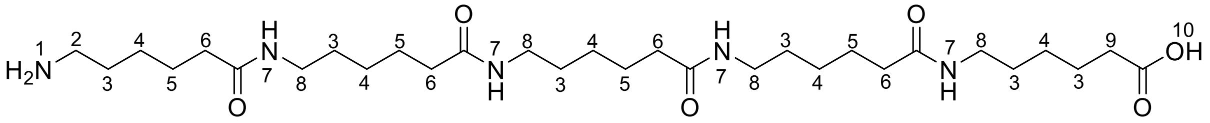

PA6$_5$

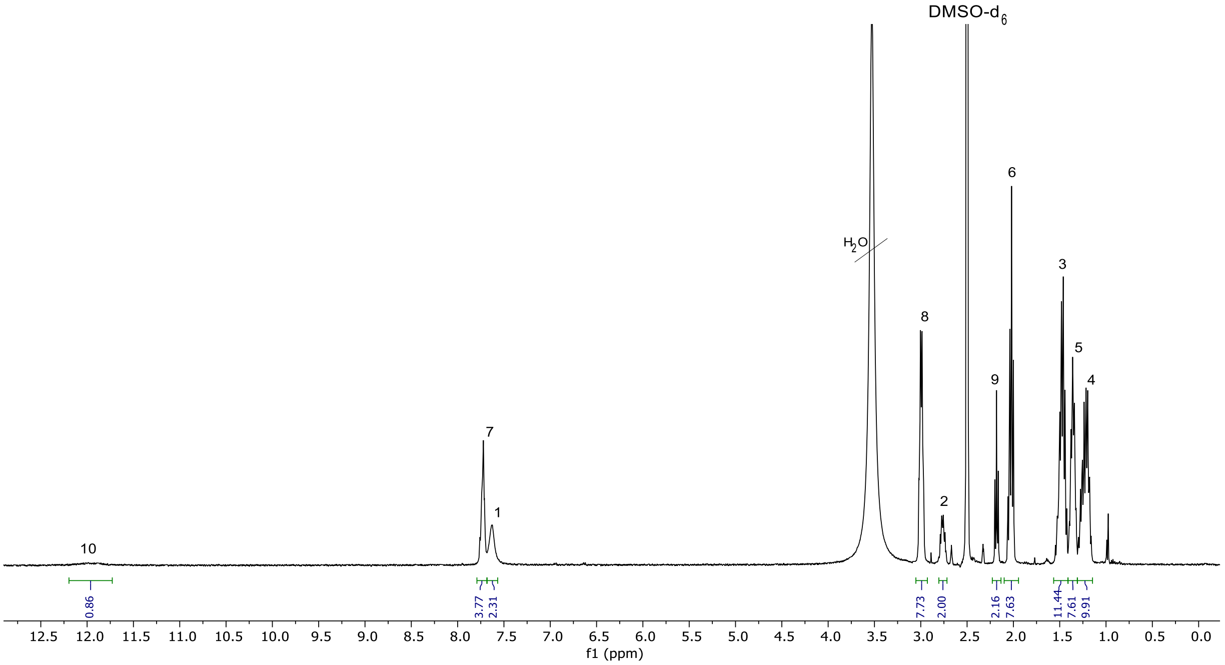

Figure S19. ^{1}H-NMR spectrum of **PA6$_5$** (400 MHz, DMSO-d$_6$).

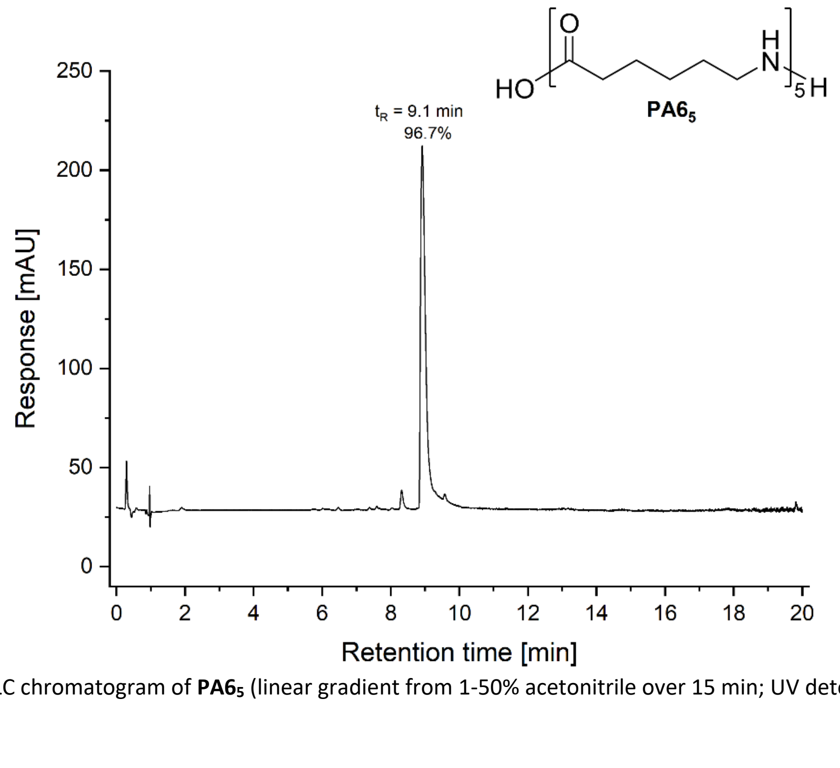

Figure S20. RP-HPLC chromatogram of **PA6$_5$** (linear gradient from 1-50% acetonitrile over 15 min; UV detection at 205 nm).

Nylon-6 Hexamer (PA6$_6$)

^{1}H-NMR (400 MHz, DMSO-d$_6$): δ (ppm) = 11.99 (br s, 1H, H-10), 7.73 (q, 5H, H-7), 7.63 (br s, 2H, H-1), 2.99 (dq, 10H, H-8), 2.82 – 2.70 (m, 2H, H-2), 2.18 (t, 2H, H-9), 2.08 – 1.98 (m, 10H, H-6), 1.57 – 1.41 (m, 14H, H-3), 1.41 – 1.31 (m, 10H, H-5), 1.30 – 1.13 (m, 12H, H-4). ESI-MS: *m/z* calcd. for $C_{36}H_{68}N_6O_7$ [M+H]$^+$ 697.5; found 697.6.

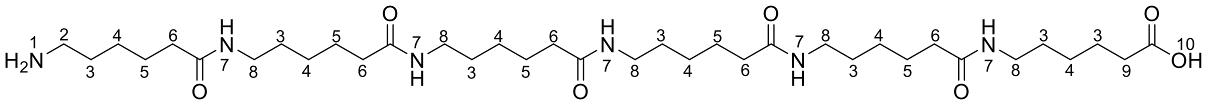

PA6$_6$

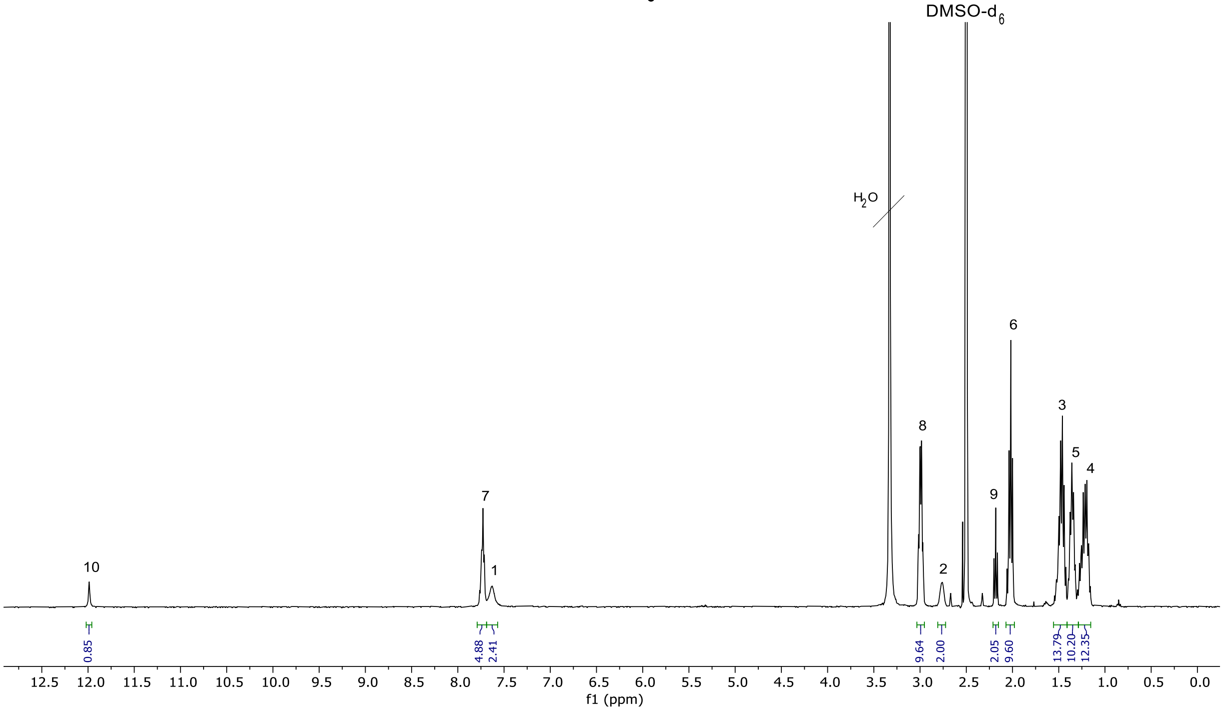

Figure S21. ^{1}H-NMR spectrum of **PA6$_6$** (400 MHz, DMSO-d$_6$).

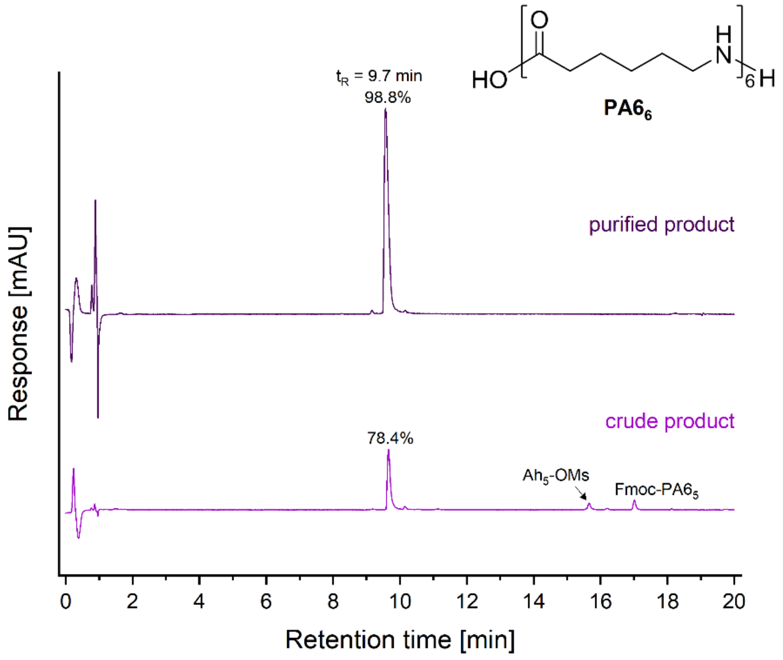

Figure S22. RP-HPLC chromatograms of **PA6$_6$** before (crude product) and after purification by preparative C18 chromatography (linear gradient from 1-50% acetonitrile over 15 min; UV detection at 205 nm).

Nylon-6 Heptamer (PA6$_7$)

Note: highly hydrogen-bonded PA6 oligomers with chain length ≥ 7 required the addition of deuterated formic acid (approx. 10% v/v) to DMSO-d$_6$ to achieve complete dissolution. Under these conditions, spectra were well-resolved and no degradation was observed within the measurement timeframe.

^{1}H-NMR (400 MHz, DMSO-d$_6$ with 10% formic acid-d$_2$): δ (ppm) = 12.64 (br s, H1;H-10), 2.99 (dt, 12H, H-8), 2.75 (t, 2H, H-2), 2.17 (t, 2H, H-9), 2.02 (t, 12H, H-6), 1.58 – 1.41 (m, 16H, H-3), 1.35 (p, 12H, H-5), 1.30 – 1.13 (m, 14H, H-4). ESI-MS: *m/z* calcd. for $C_{42}H_{79}N_7O_8$ [M+H]$^+$ 810.6; found 810.8.

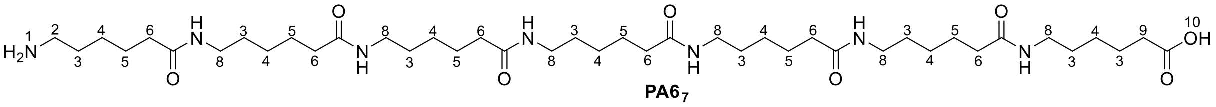

PA6$_7$

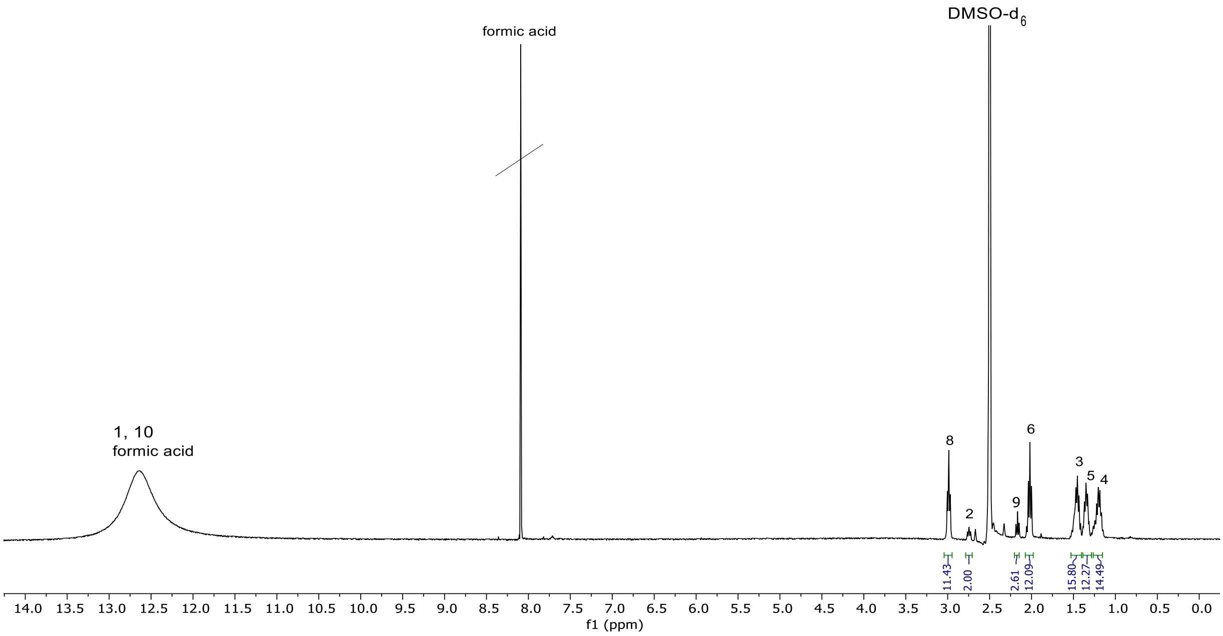

Figure S23. ^{1}H-NMR spectrum of **PA6$_7$** (400 MHz, DMSO-d$_6$ + 10% formic acid-d$_2$). Samples required the addition of deuterated formic acid (≈ 10% v/v) in DMSO-d$_6$ to achieve complete dissolution.

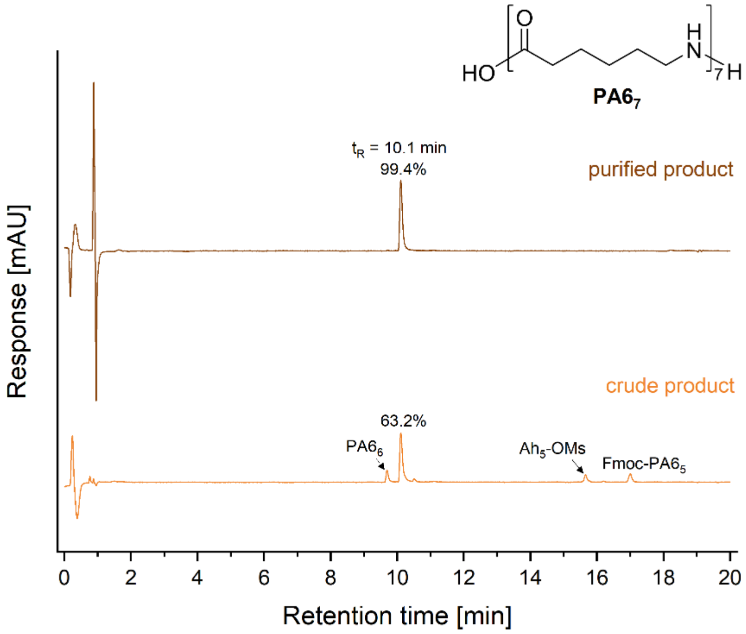

Figure S24. RP-HPLC chromatograms of **PA6$_7$** before (crude product) and after purification by preparative C18 chromatography (linear gradient from 1-50% acetonitrile over 15 min; UV detection at 205 nm).

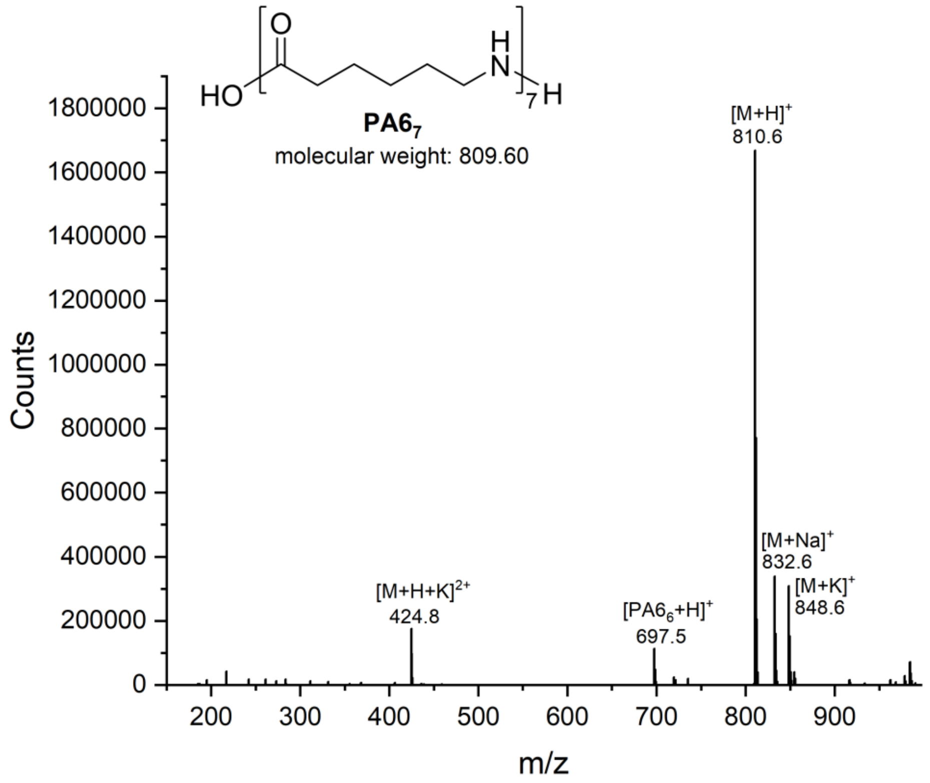

Figure S25. ESI mass spectrum of **PA6$_7$** recorded in HFIP.

Nylon-6,6 Dimer (PA66$_2$)

^{1}H-NMR (400 MHz, DMSO-d$_6$): δ (ppm) = 11.99 (br s, 1H, H-8), 7.73 (q, 3H, H-5), 7.63 (br s, 2H, H-1), 3.00 (p, 6H, H-4), 2.81 – 1.98 (m, 2H, H-2), 2.19 (t, 2H, H-7), 2.07 – 1.98 (m, 6H, H-6), 1.53 – 1.17 (m, 24H, H-3). ESI-MS: *m/z* calcd. for $C_{24}H_{46}N_4O_5$ [M+H]$^+$ 471.4; found 471.4.

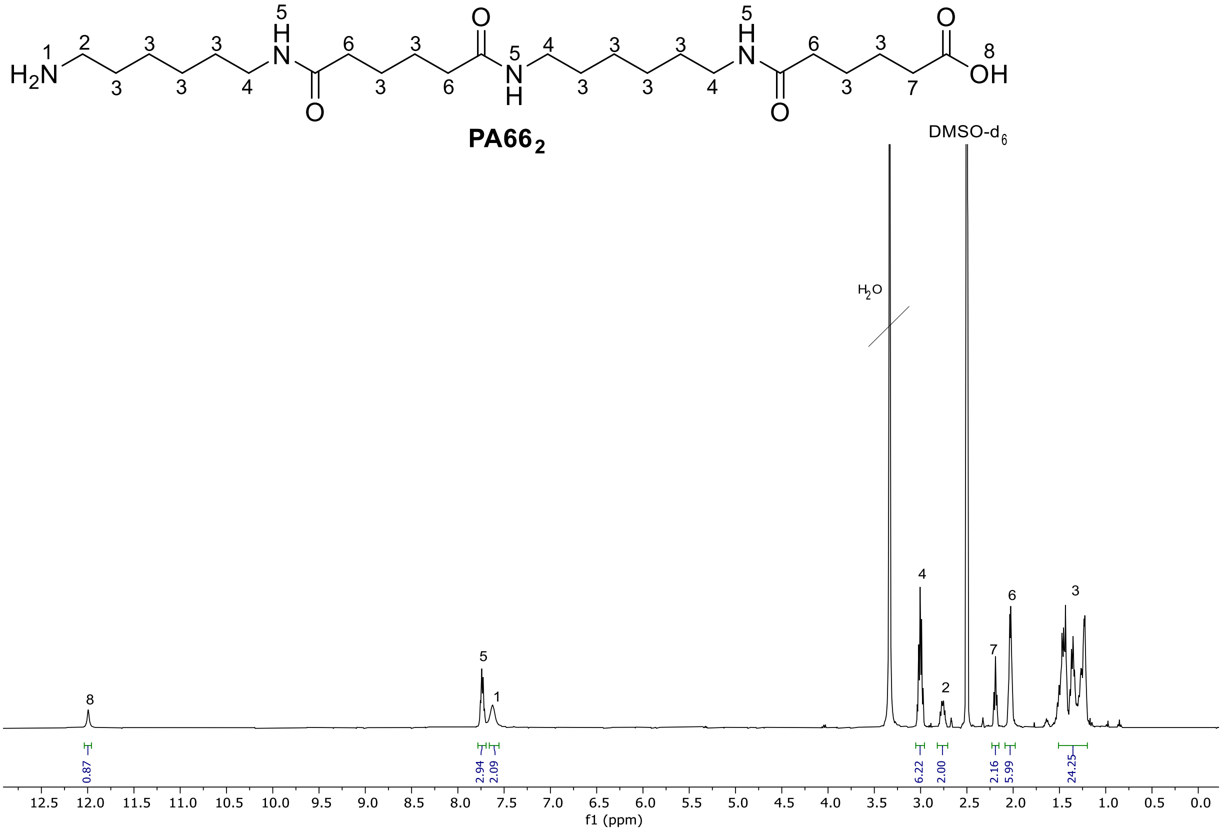

Figure S26. ^{1}H-NMR spectrum of **PA66$_2$** (400 MHz, DMSO-d$_6$).

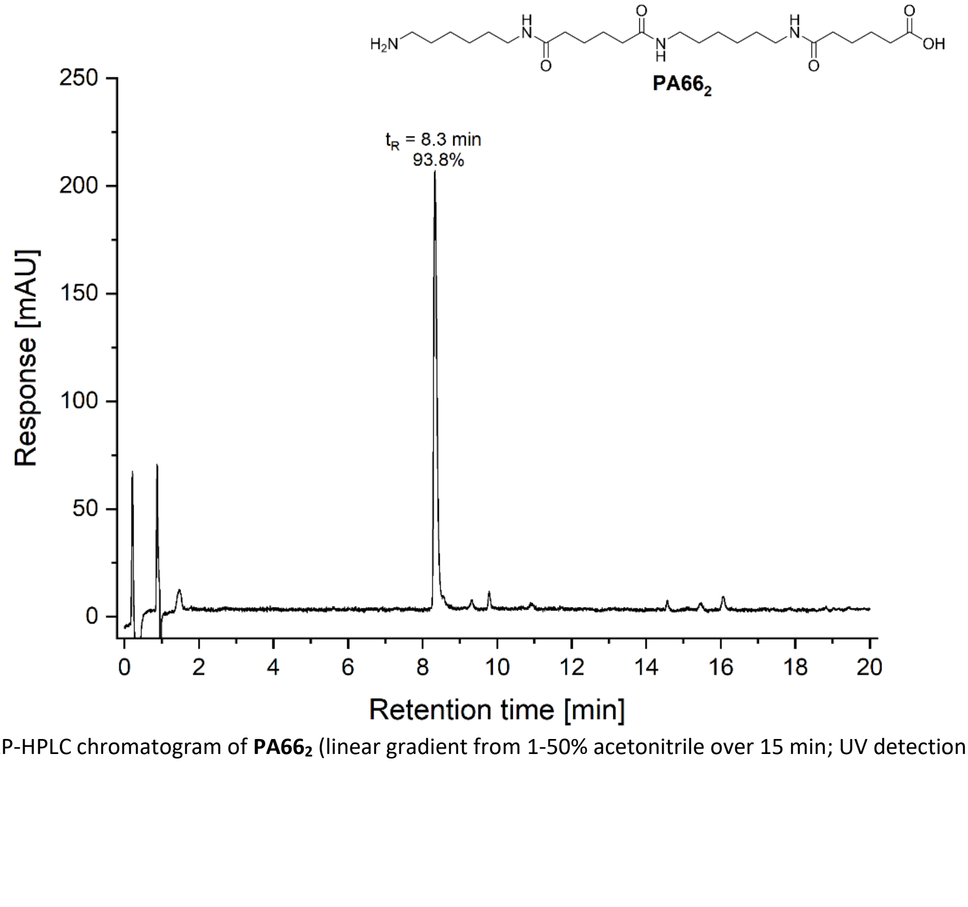

Figure S27. RP-HPLC chromatogram of **PA66$_2$** (linear gradient from 1-50% acetonitrile over 15 min; UV detection at 205 nm).

Nylon-6,6 Trimer (PA66$_3$)

^{1}H-NMR (400 MHz, DMSO-d$_6$): δ (ppm) = 11.99 (br s, 1H, H-8), 7.73 (q, 5H, H-5), 7.61 (br s, 2H, H-1), 3.00 (p, 10H, H-4), 2.81 – 2.72 (m, 2H, H-2), 2.19 (t, 2H, H-7), 2.08 – 1.99 (m, 10H, H-6), 1.61 – 1.11 (m, 36H, H-3). ESI-MS: *m/z* calcd. for $C_{36}H_{68}N_6O_7$ [M+H]$^+$ 697.9; found 697.8.

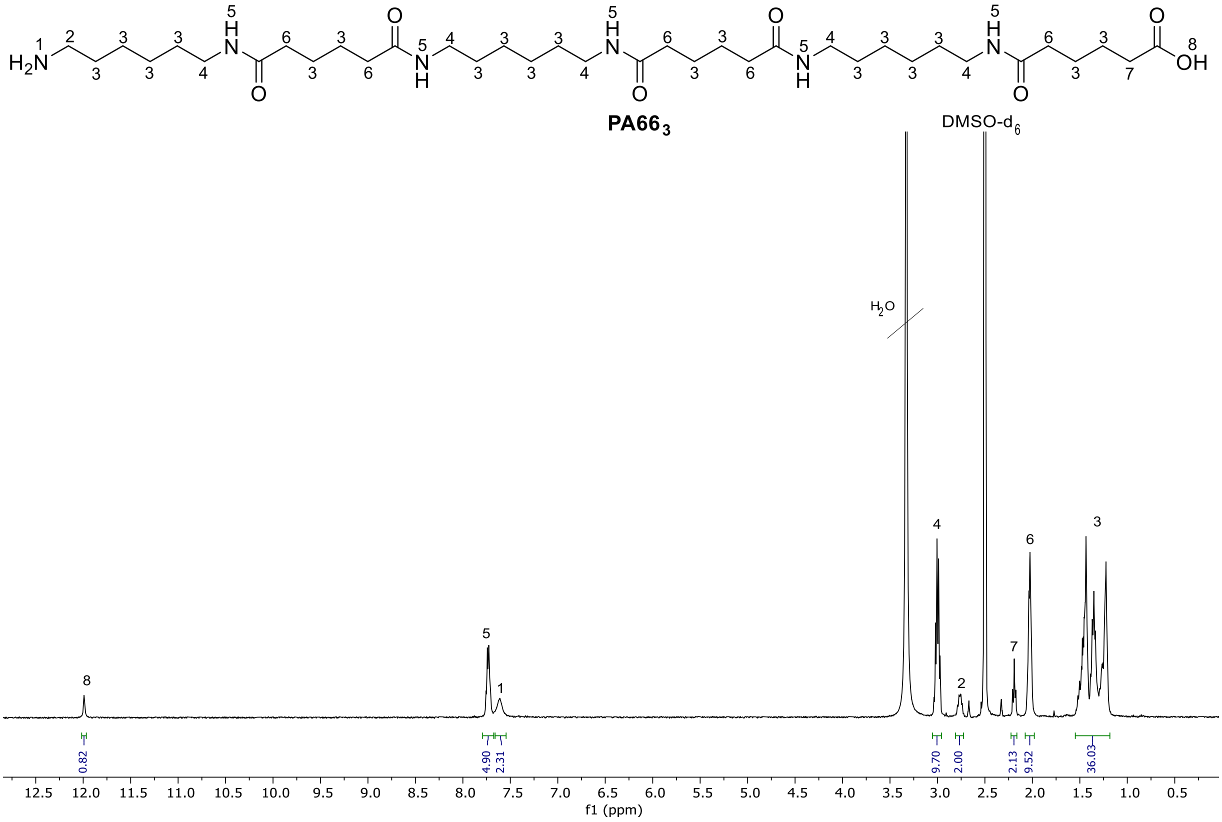

Figure S28. ^{1}H-NMR spectrum of **PA66$_3$** (400 MHz, DMSO-d$_6$).

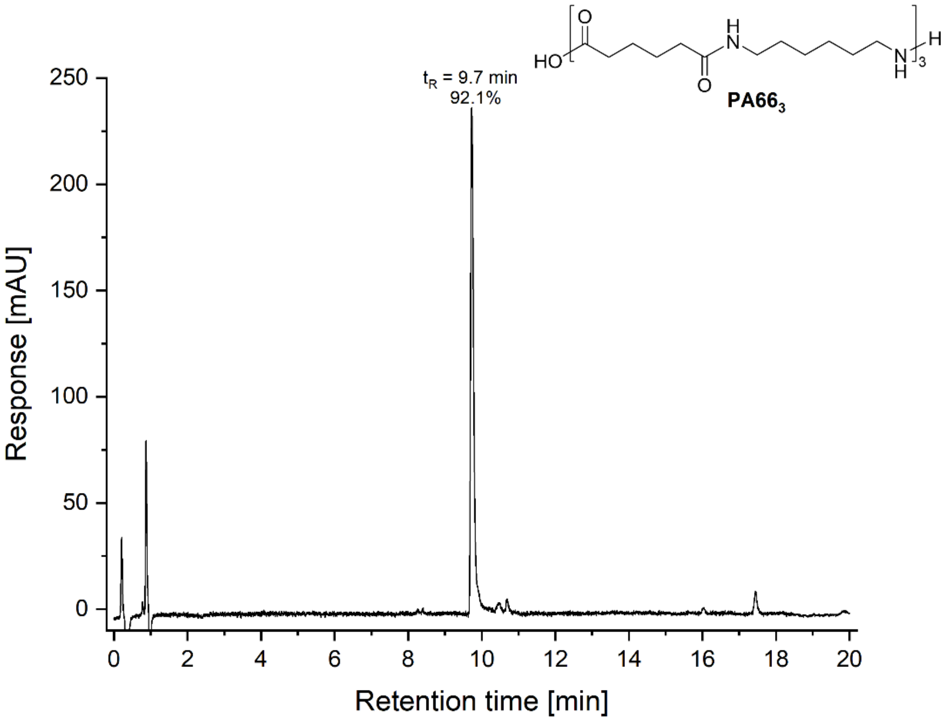

Figure S29. RP-HPLC chromatogram of **PA66$_3$** (linear gradient from 1-50% acetonitrile over 15 min; UV detection at 205 nm).

Nylon-6,6 Tetramer (PA66$_4$)

Note: highly hydrogen-bonded PA66 oligomers with chain length ≥ 4 required the addition of deuterated formic acid (approx. 10% v/v) to DMSO-d$_6$ to achieve complete dissolution. Under these conditions, spectra were well-resolved and no degradation was observed within the measurement timeframe.

^{1}H-NMR (400 MHz, DMSO-d$_6$ with 10% formic acid-d$_2$): δ (ppm) = 12.25 (br s, H-1;H-8), 3.00 (q, 14H, H-4), 2.81 – 2.72 (m, 2H, H-2), 2.18 (t, 2H, H-7), 2.08 – 1.99 (m, 14H, H-6), 1.56 – 1.16 (m, 48H, H-3).ESI-MS: *m/z* calcd. for $C_{48}H_{90}N_8O_9$ $[M+H]^+$ 923.7; found 923.6.

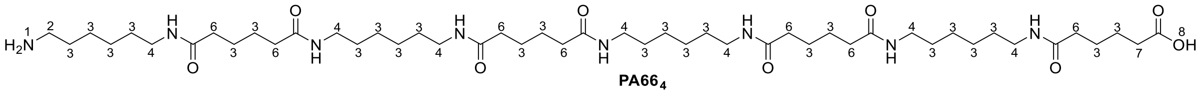

PA66$_4$

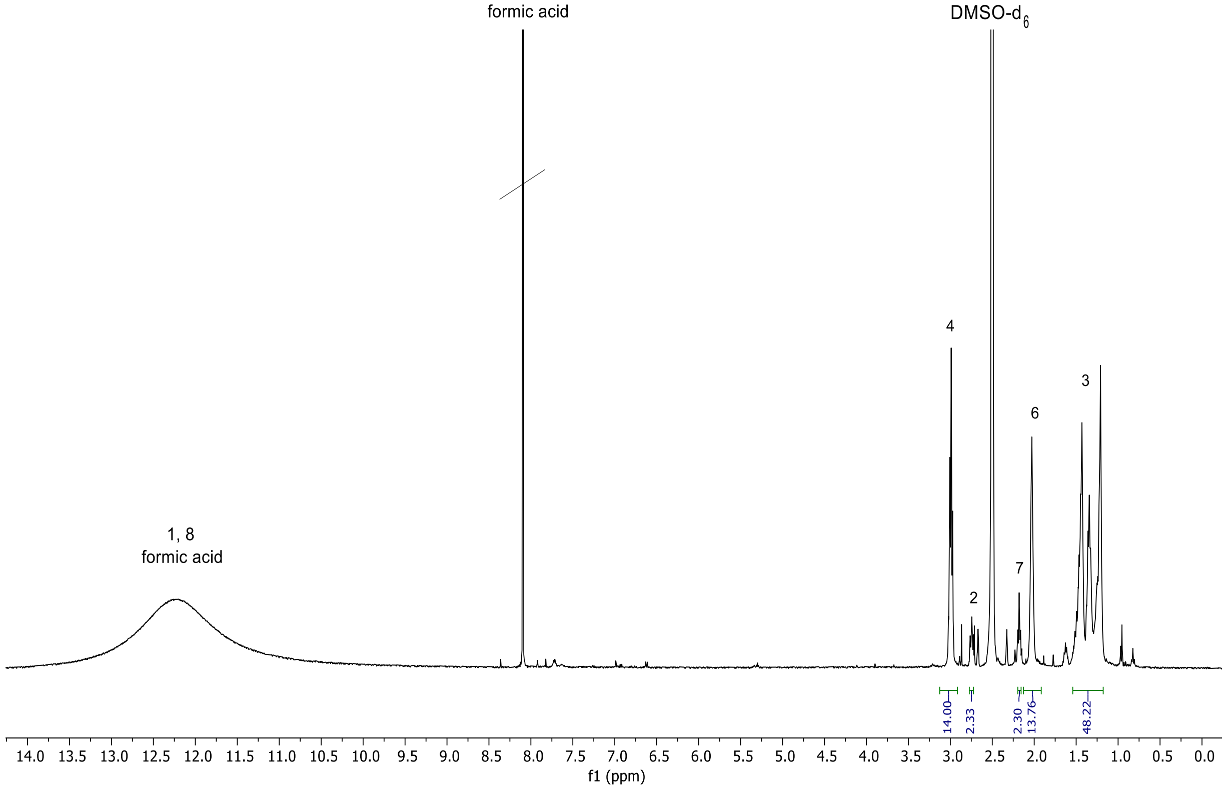

Figure S30. ^{1}H-NMR spectrum of **PA66$_4$** (400 MHz, DMSO-d$_6$).

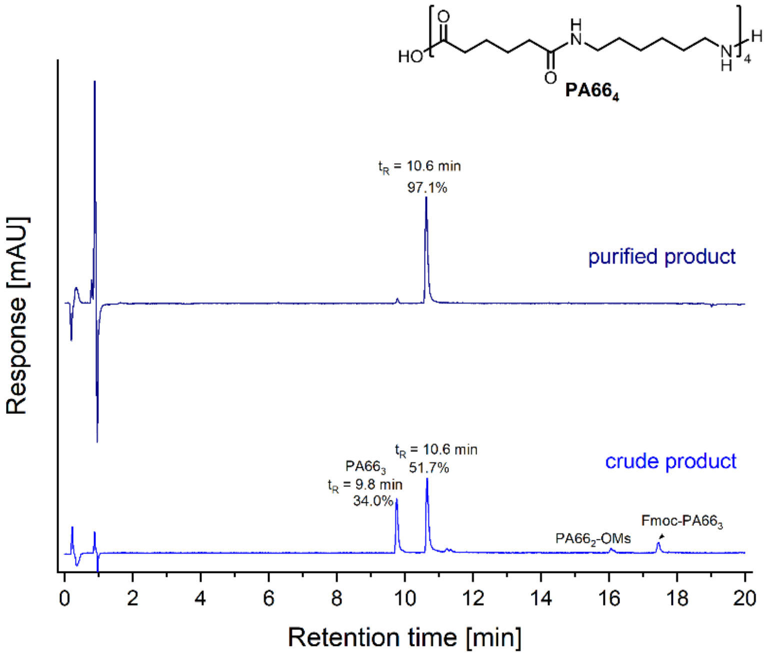

Figure S31. RP-HPLC chromatograms of **PA66$_4$** before (crude product) and after purification by preparative C18 chromatography (linear gradient from 1-50% acetonitrile over 15 min; UV detection at 205 nm).

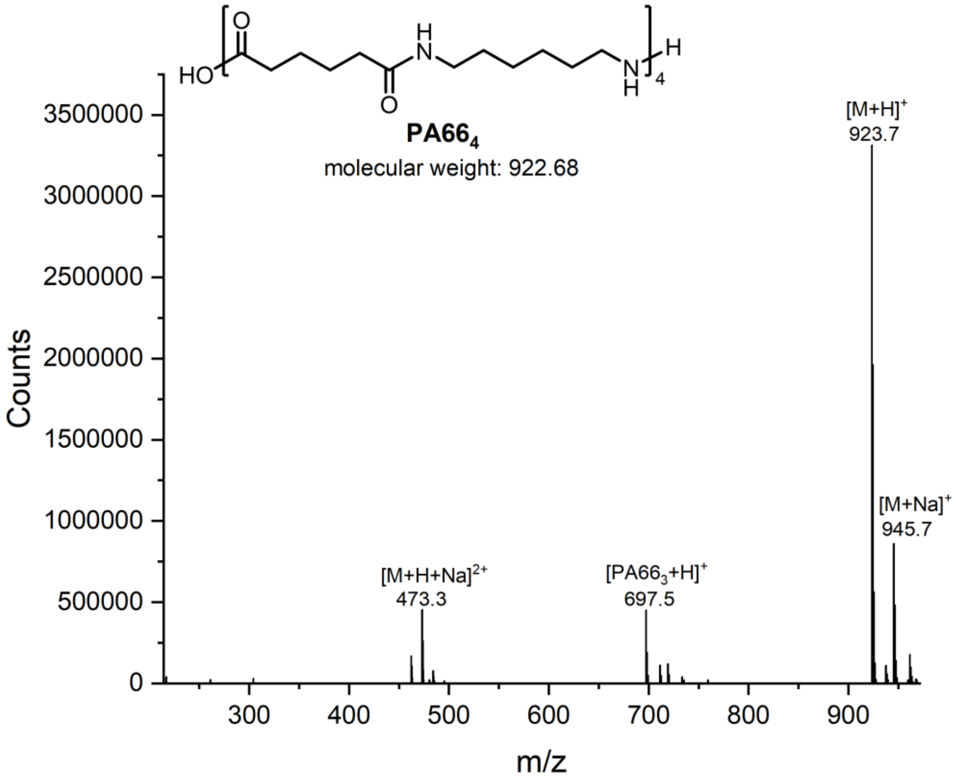

Figure S32. ESI mass spectrum of **PA66$_4$** recorded in HFIP.

Enzyme Expression and Purification

Enzyme expression and purification were carried out according to the method described by Puetz *et al*.[5] the selected NylC variant, NylC-HP ($NylC_{p2}$-$TS^{F134W/D304M/R330A}$), was expressed in *Escherichia coli* BL21(DE3). Cultures were grown at 37 °C to an OD_{600} of ~0.6 and induced with 0.1 mM IPTG. Protein expression proceeded for 20 h at 18 °C. Cells were harvested by centrifugation (3,220 × g, 20 min, 4 °C) and resuspended in lysis buffer (50 mM NaH_2PO_4, pH 8.0, 300 mM NaCl) supplemented with lysozyme (1.5 mg mL^{-1}). After incubation for 30 min at 37 °C, cells were lysed by sonication (Vibra-Cell VCX130, Sonics & Materials Inc., USA; 5 × 30 s pulses at 60% amplitude with 30 s cooling intervals).

The lysate was clarified by centrifugation (10,000 rpm, 30 min, 4 °C; Eppendorf 5810R, Hamburg, Germany), filtered through a 0.45 µm syringe filter, and applied to a Ni–IDA 2000 affinity column (Macherey-Nagel GmbH, Düren, Germany). The column was washed with 30 mL lysis buffer, and NylC-HP was eluted with 2.5 mL elution buffer (50 mM NaH_2PO_4, pH 8.0, 300 mM NaCl, 250 mM imidazole).

The elution buffer was exchanged for storage buffer (50 mM Bicine, pH 8.0, 100 mM NaCl) using PD-10 desalting columns (Cytiva, MA, USA) and the protein was concentrated with 10 kDa MWCO Amicon Ultra centrifugal filters (Merck, Darmstadt, Germany). Purified NylC-HP was snap-frozen in liquid nitrogen and stored at -20 °C. Protein purity was confirmed by SDS-PAGE, and concentrations were determined using the Bradford assay (Pierce Coomassie Plus, Thermo Fisher Scientific, Wesel, Germany).[6]

Enzymatic Hydrolysis of Nylon-6 and Nylon-6.6

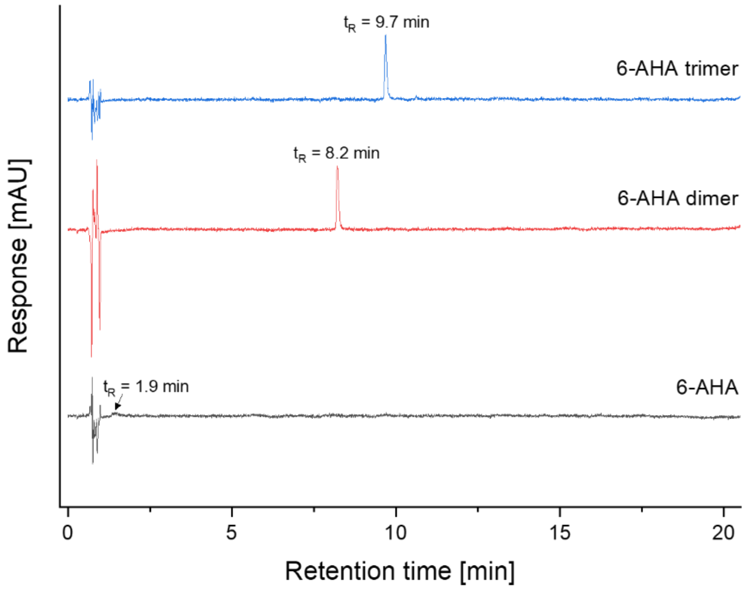

Figure S33. RP-HPLC method of PA6 oligomer degradation products. Chromatograms of 6-aminohexanoic acid (6-AHA) monomer (retention time 1.9 min), 6-AHA dimer (8.2 min), and 6-AHA trimer (9.7 min), each at 0.1 mM. Chromatograms were recorded using an isocratic hold at 100% water for 3 min, followed by a linear gradient from 0-50% acetonitrile over 15 min with UV detection at 205 nm.

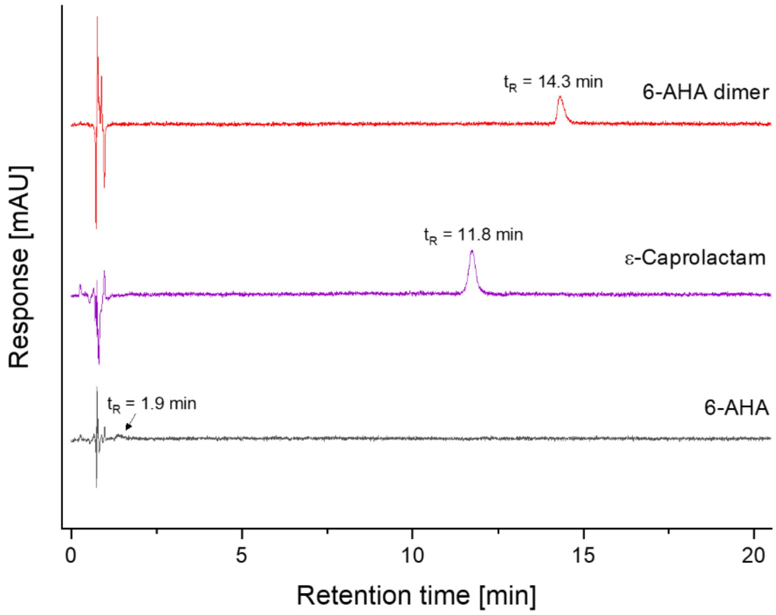

Figure S34. RP-HPLC method of PA6 film degradation products. Chromatograms of 6-aminohexanoic acid (6-AHA) monomer (retention time 1.9 min), ε-caprolactam (11.8 min), and 6-AHA dimer (14.3 min), each at 0.1 mM. Chromatograms were recorded using an isocratic hold at 100% water for 5 min, followed by a linear gradient from 0-10% acetonitrile over 15 min with UV detection at 205 nm.

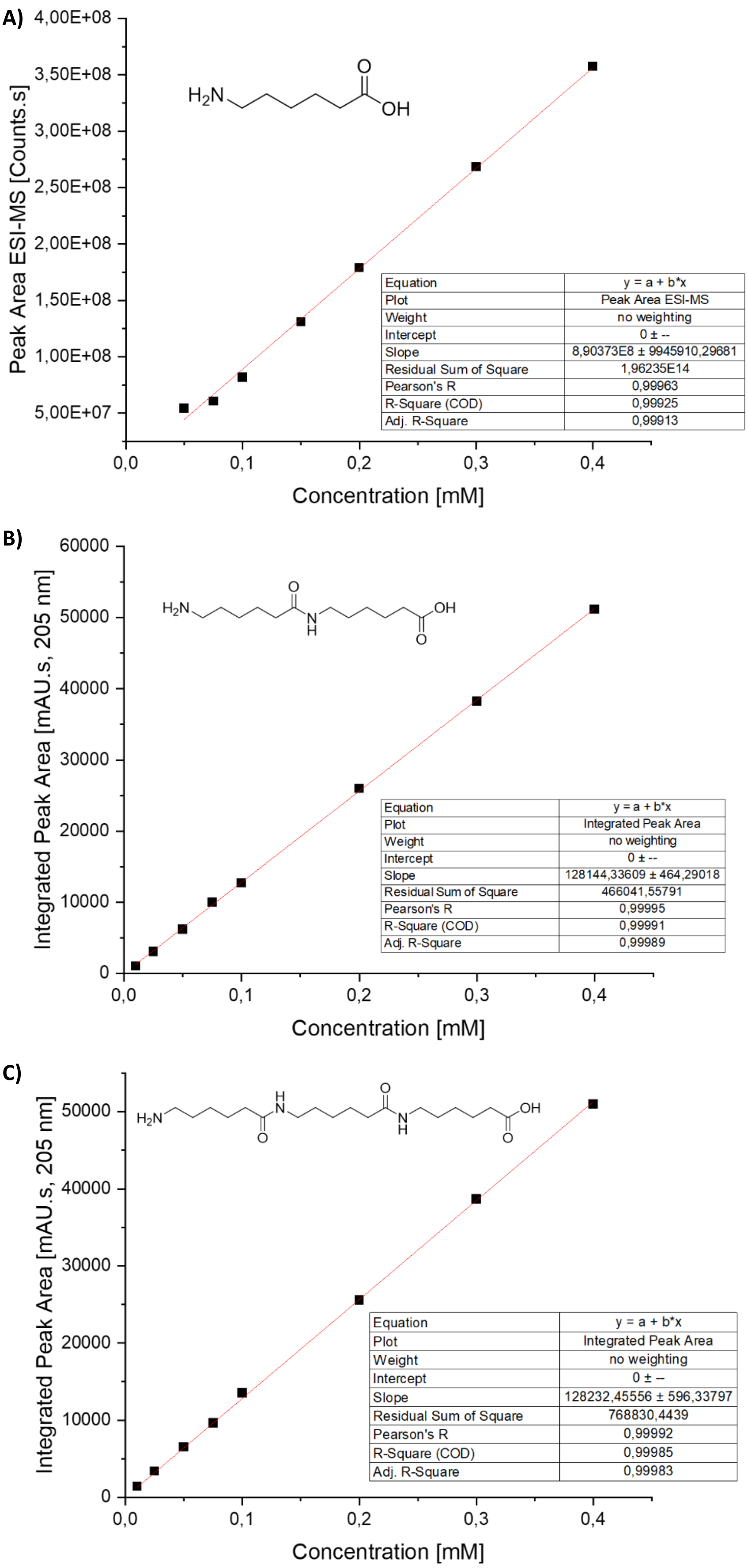

Figure S35. Standard curves for RP-HPLC analysis of the PA6 oligomer degradation assay. A) 6-aminohexanoic acid (6-AHA) ESI-MS peak area. B) 6-AHA dimer UV peak area (205 nm). C) 6-AHA trimer UV peak area (205 nm). Chromatograms were recorded using an isocratic hold at 100% water for 3 min, followed by a linear gradient from 0-50% acetonitrile over 15 min.

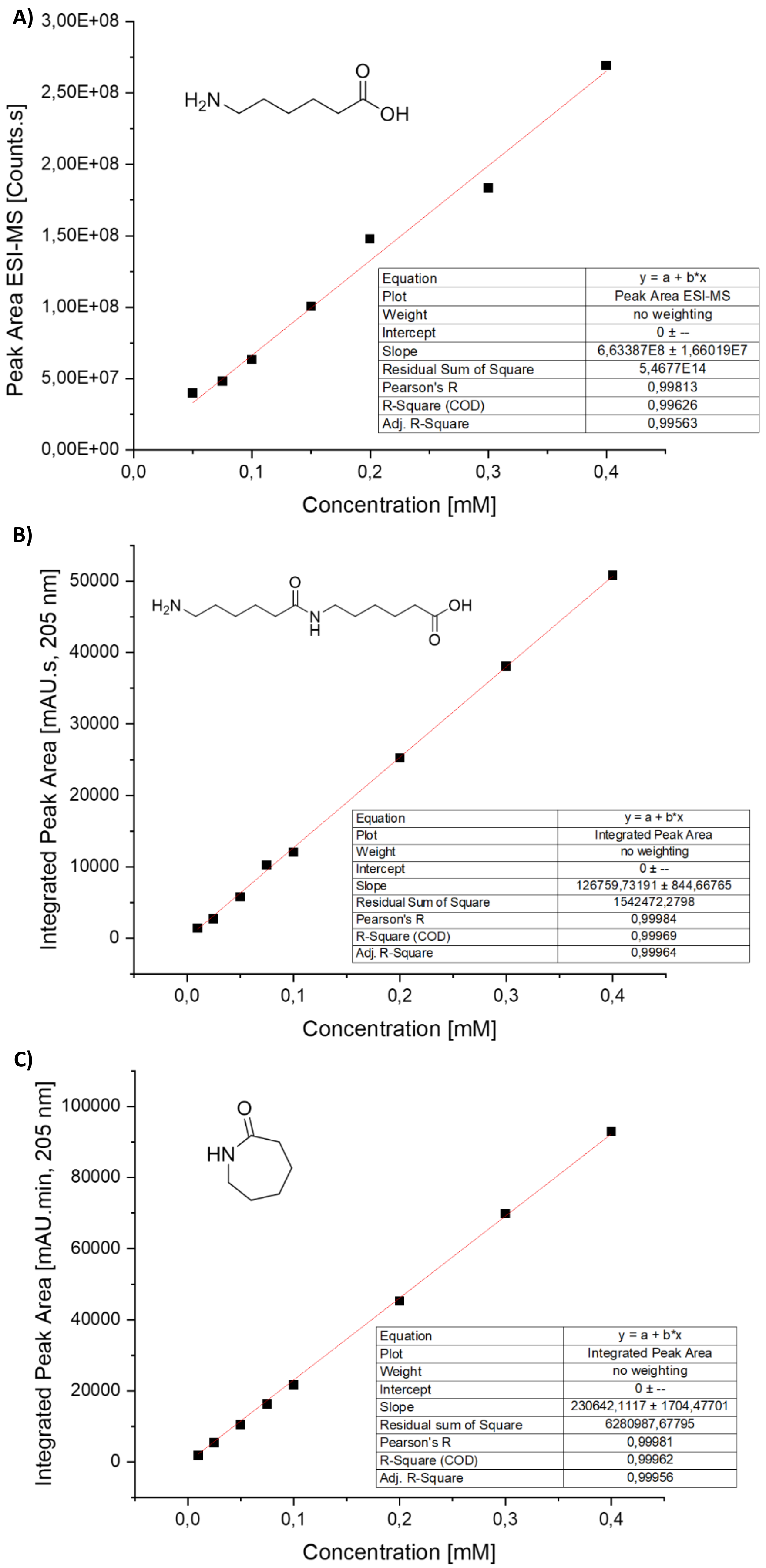

Figure S36. Standard curves for RP-HPLC analysis of the PA6 film degradation assay. A) 6-aminohexanoic acid (6-AHA) ESI-MS peak area. B) 6-AHA dimer UV peak area (205 nm). C) ε-Caprolactam UV peak area (205 nm). Chromatograms were recorded using an isocratic hold at 100% water for 5 min, followed by a linear gradient from 0-10% acetonitrile over 15 min.

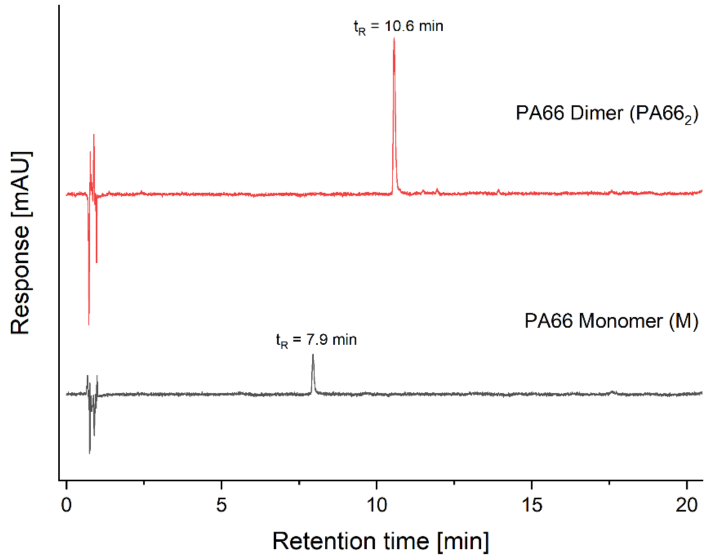

Figure S37. RP-HPLC method of PA66 degradation products. Chromatograms of PA66 monomer M (retention time 7.9 min) and PA66 dimer (10.6 min), each at 0.1 mM. Chromatograms were recorded using an isocratic hold at 100% water for 3 min, followed by a linear gradient from 0-50% acetonitrile over 15 min with UV detection at 205 nm.

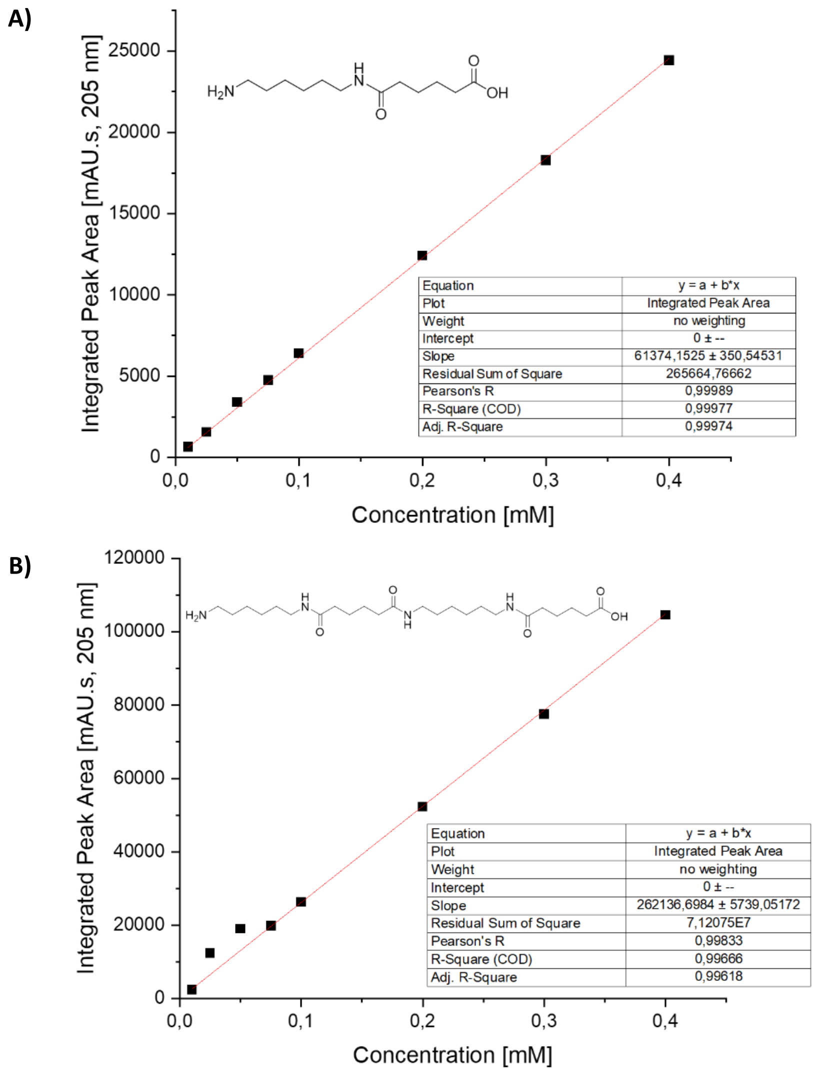

Figure S38. Standard curves for RP-HPLC analysis of the PA66 degradation assay. A) PA66 monomer (M) UV peak area. B) PA66 dimer UV peak area. The cyclic monomer (cy-Mono) and the diacid-terminated product (M–AA) were quantified using the calibration curve of M as a surrogate standard, whereas the cyclic dimer (cy-Dimer) was quantified using the calibration curve of the PA66 dimer. Chromatograms were recorded using an isocratic hold at 100% water for 3 min, followed by a linear gradient from 0-50% acetonitrile over 15 min with UV detection at 205 nm.

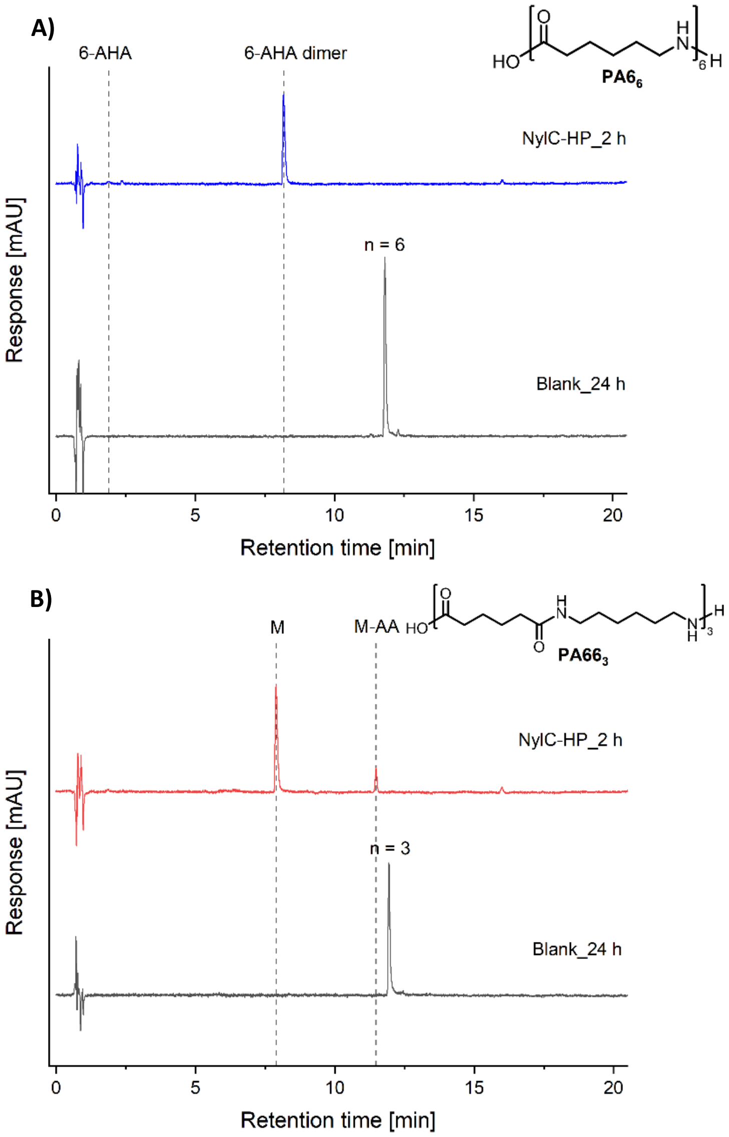

Figure S39. RP-HPLC chromatograms comparing enzyme-treated and control reactions: A) PA6 hexamer ($PA6_6$) and B) PA66 trimer ($PA66_3$) after incubation at 70 °C. Chromatograms were recorded using an isocratic hold at 100% water for 3 min, followed by a linear gradient from 0-50% acetonitrile over 15 min with UV detection at 205 nm.

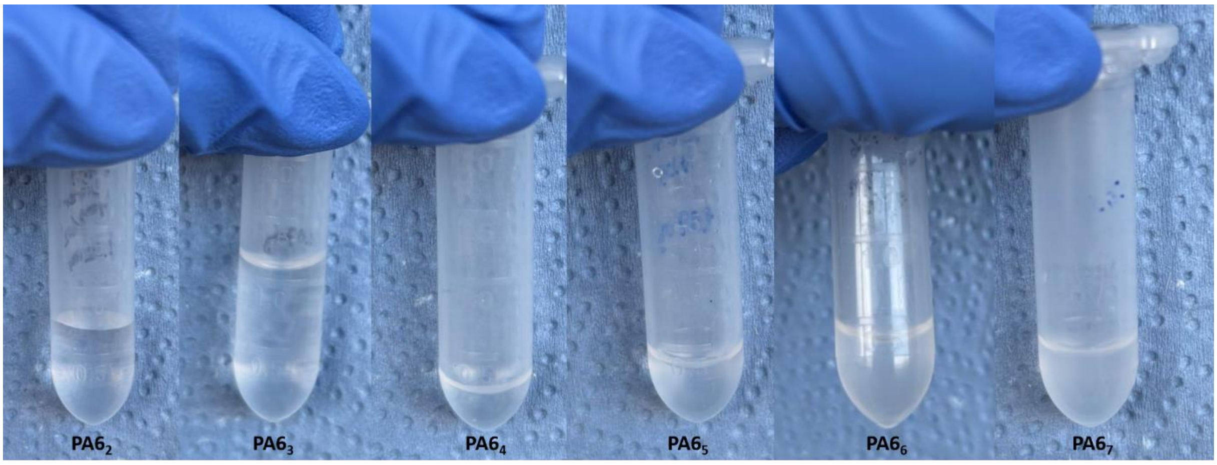

Figure S40. Qualitative solubility assessment of defined PA6 oligomers (dimer to heptamer) showing the transition from fully dissolved to dispersed states with increasing chain length.

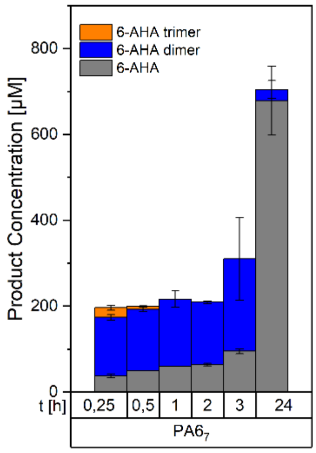

Figure S41. Time-resolved product formation from PA6 heptamer (100 mM) by NylC-HP at 70 °C. Reactions were performed in triplicate (n = 3); error bars represent the standard deviation of replicate measurements.

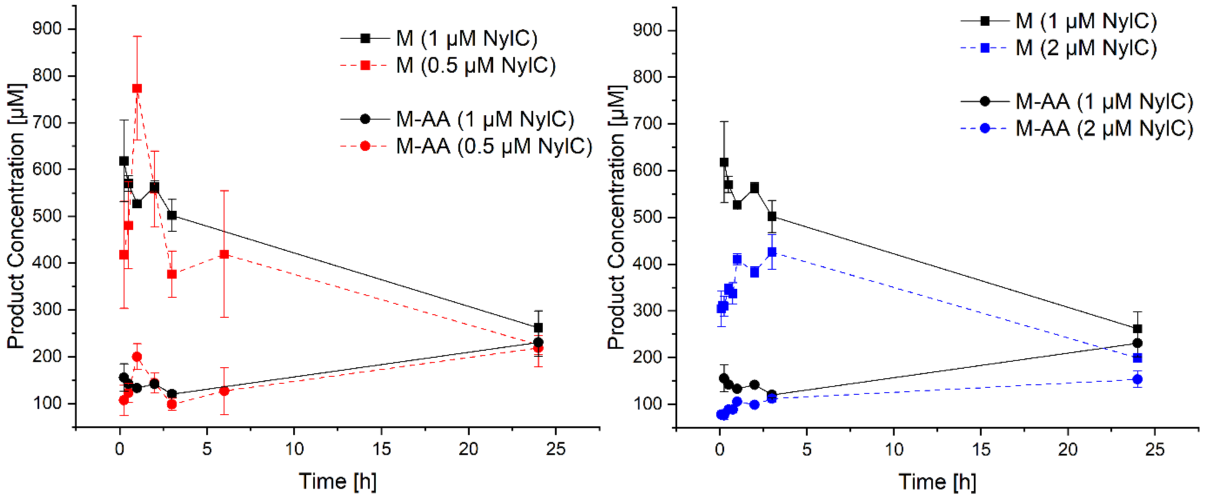

Figure S42. Enzyme-scaling experiment for the hydrolysis of $PA66_4$ by NylC-HP at 70 °C. The effect of enzyme concentration (0.5–2 µM) on product formation and distribution of the monomer (M) and the diacid fragment (M–AA) over time. Reactions were performed in duplicate (n = 2); error bars represent the standard deviation of replicate measurements.

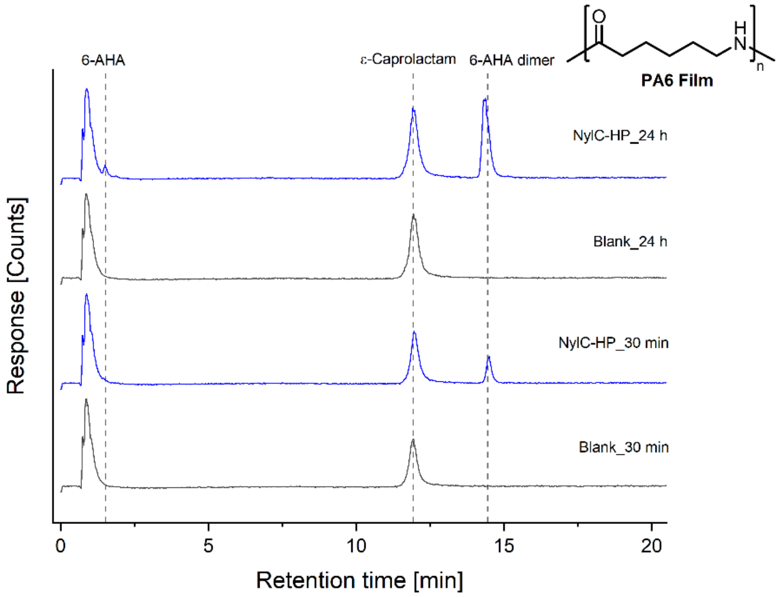

Figure S43. ESI-MS extracted-ion chromatograms (EICs) for PA6 film incubated without enzyme (blank, black) or with NylC-HP (blue) at 70 °C. Chromatograms were recorded using an isocratic hold at 100% water for 5 min, followed by a 0-10% acetonitrile gradient over 15 min.

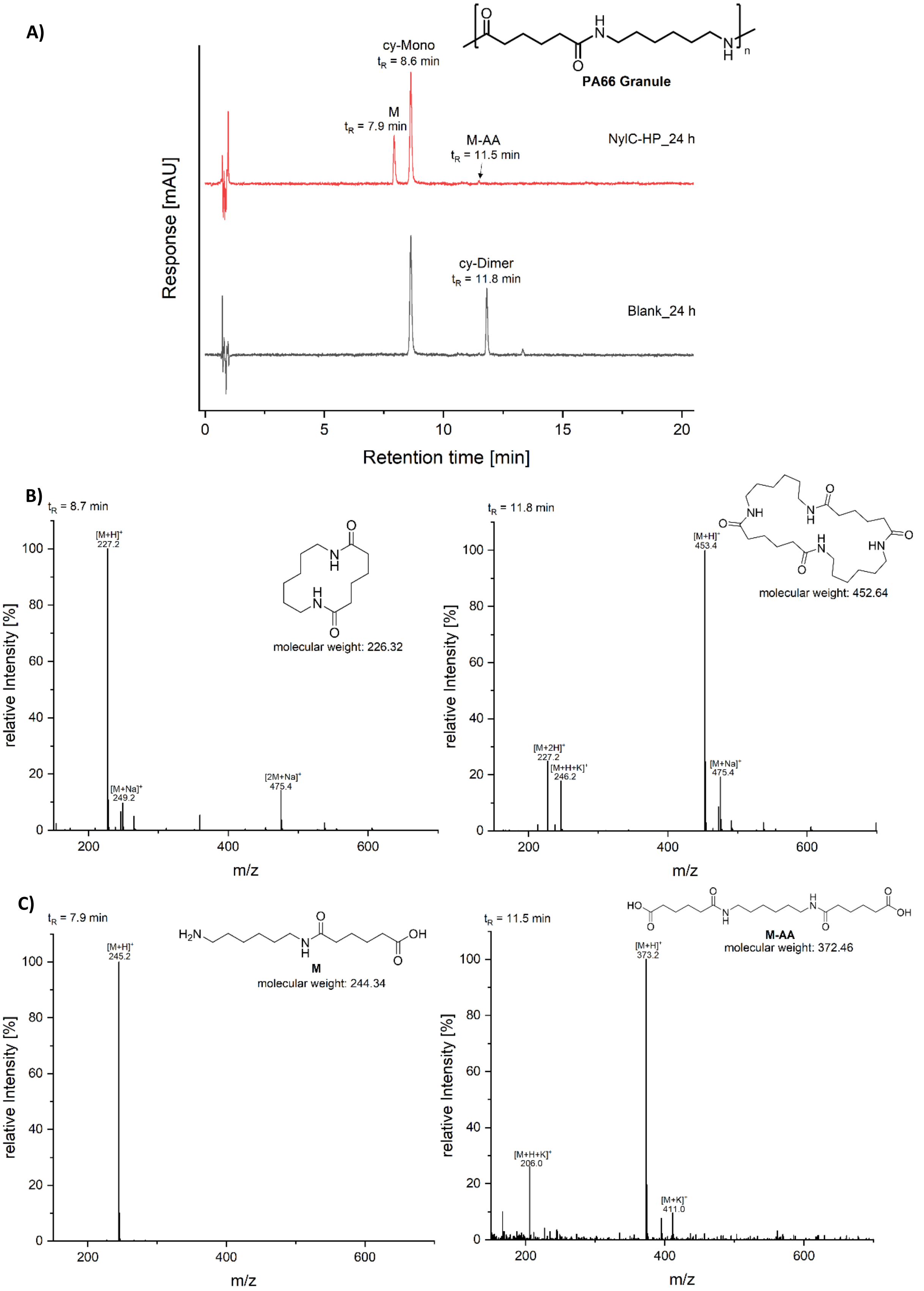

Figure S44. A) RP-HPLC chromatograms of PA66 granules incubated without enzyme (blank, black) or with NylC-HP (red) at 70 °C for 24 h. Cyclic monomer (cy-Mono), cyclic dimer (cy-Dimer), the linear monomeric product M (HMDA-AA), and diacid-terminated fragment M−AA are indicated. Chromatograms were recorded using an isocratic hold at 100% water for 3 min followed by a linear gradient from 0-50% acetonitrile over 15 min with UV detection at 205 nm. B) ESI-MS spectra of the cyclic monomer (left) and cyclic dimer (right) detected in blank samples. C) ESI-MS spectra of the linear hydrolysis products M (left) and M−AA (right) formed in enzyme-treated samples.

References

[1] D. Ponader, F. Wojcik, F. Beceren-Braun, J. Dernedde, L. Hartmann, *Biomacromolecules* **2012**, *13*, 1845–1852.

[2] M. F. Ebbesen, C. Gerke, P. Hartwig, L. Hartmann, *Polym. Chem.* **2016**, *7*, 7086–7093.

[3] S. Boden, K. G. Wagner, M. Karg, L. Hartmann, *Polymers* **2017**, *9*.

[4] C. Robert, F. de Montigny, C. M. Thomas, *ACS Catal.* **2014**, *4*, 3586–3589.

[5] H. Puetz, A.-M. Illig, M. Vorobii et al., *ChemSusChem* **2025**, *18*, e202500257.

[6] M. M. Bradford, *Anal. Biochem.* **1976**, *72*, 248–254.
